# Antibody landscape of C57BL/6 mice cured of B78 melanoma via a combined radiation and immunocytokine immunotherapy regimen

**DOI:** 10.3389/fimmu.2023.1221155

**Published:** 2023-11-23

**Authors:** Anna Hoefges, Sean J. McIlwain, Amy K. Erbe, Nicholas Mathers, Angie Xu, Drew Melby, Kaitlin Tetreault, Trang Le, Kyungmann Kim, Richard S. Pinapati, Bradley H. Garcia, Jigar Patel, Mackenzie Heck, Arika S. Feils, Noah Tsarovsky, Jacquelyn Ann Hank, Zachary Scott Morris, Irene M. Ong, Paul Mark Sondel

**Affiliations:** ^1^ Department of Human Oncology, University of Wisconsin, Madison, WI, United States; ^2^ Department of Biostatistics and Medical Informatics, University of Wisconsin, Madison, WI, United States; ^3^ Nimble Therapeutics, Inc., Madison, WI, United States; ^4^ Department of Obstetrics and Gynecology, University of Wisconsin, Madison, WI, United States; ^5^ Department of Pediatrics, University of Wisconsin, Madison, WI, United States

**Keywords:** high-density peptide array, melanoma, *in situ* vaccine, radio-immunotherapy, antibody, cancer, proteome

## Abstract

Sera of immune mice that were previously cured of their melanoma through a combined radiation and immunocytokine immunotherapy regimen consisting of 12 Gy of external beam radiation and the intratumoral administration of an immunocytokine (anti-GD2 mAb coupled to IL-2) with long-term immunological memory showed strong antibody-binding against melanoma tumor cell lines via flow cytometric analysis. Using a high-density whole-proteome peptide array (of 6.090.593 unique peptides), we assessed potential protein-targets for antibodies found in immune sera. Sera from 6 of these cured mice were analyzed with this high-density, whole-proteome peptide array to determine specific antibody-binding sites and their linear peptide sequence. We identified thousands of peptides that were targeted by these 6 mice and exhibited strong antibody binding only by immune (after successful cure and rechallenge), not naïve (before tumor implantation) sera and developed a robust method to detect these differentially targeted peptides. Confirmatory studies were done to validate these results using 2 separate systems, a peptide ELISA and a smaller scale peptide array utilizing a slightly different technology. To the best of our knowledge, this is the first study of the full set of germline encoded linear peptide-based proteome epitopes that are recognized by immune sera from mice cured of cancer via radio-immunotherapy. We furthermore found that although the generation of B-cell repertoire in immune development is vastly variable, and numerous epitopes are identified uniquely by immune serum from each of these 6 immune mice evaluated, there are still several epitopes and proteins that are commonly recognized by at least half of the mice studied. This suggests that every mouse has a unique set of antibodies produced in response to the curative therapy, creating an individual “fingerprint.” Additionally, certain epitopes and proteins stand out as more immunogenic, as they are recognized by multiple mice in the immune group.

## 1 Introduction

Cancer immunotherapy has revolutionized cancer treatment and has helped thousands of patients (1, 2). However, most patients are still not showing positive responses to current cancer immunotherapy treatment regimens (2, 3). Using radiation therapy (RT) and intratumoral injections of immunocytokine (IC), we have developed a local *in-situ* vaccine (ISV, RT+IC) regimen capable of curing immunocompetent C57BL/6 mice bearing syngeneic B78 melanoma tumors and resulting in protective immune memory (4). Even though B78 is considered a functionally “cold” tumor due to its lack of response to checkpoint inhibitors (5, 6), our RT+IC regimen can cure many of them. With our *in-situ* vaccine, RT acts to increase the immunogenicity of the tumor by modifying its phenotype and releasing immune stimulatory cytokines. The IC used here is an engineered fusion protein consisting of a tumor-specific monoclonal antibody targeting disialoganglioside (GD2) linked to IL2. GD2 is a molecule expressed on the surface of most neuroectodermal tumors and some nerve fibers. We also demonstrated that our *in-situ* vaccine causes epitope spread; 75% of cured mice reject a re-challenge with B16 melanoma cells (4, 7). B16 melanoma cells do not express the GD2 antigen and are the parental cell line to B78 (8–10). We observed strong antibody-binding to B16 cells using serum from cured as compared to naïve mice (11). These antibodies might enable MHC-independent, CD8-T cell independent anti-tumor adaptive immune responses via macrophage-mediated antibody-dependent direct tumor cell killing (12). However, the exact antigen targets of these endogenous antibodies are unknown.

Identifying epitopes on tumor cells that are recognized by antibodies may help identify the immunodominant antigens of cold human tumors, which may help in overcoming immune resistance in these cancers (13–15). With the RT+IC regimen, although we are targeting GD2, the memory response does not require GD2 presence on the tumor cells as GD2 negative melanoma cells can be rejected during rechallenge of cured mice (4). Knowledge of these additional antigenic targets that enable immune memory may help to identify biomarkers of positive responses and identify potential new therapeutic targets.

In this paper, we utilized a high-density peptide array approach to probe every protein of the mouse proteome, broken down into 16-mer peptides in a 2 or 4 amino acid (aa) tiling approach, to identify antibody targets, using serum from cured mice vs. their matched naïve sample. This high-density peptide array technology has been used for several productive applications recently (16–21). Using this approach, we identified many antigens expressed by cold murine tumors in individual mice as well as some antigens that are recognized by multiple mice. The main finding of this manuscript is that despite the immense variation within the generation of B-cell repertoire in immune development, and the large number of epitopes identified for each individual mouse, we found many epitopes and proteins co-recognized by at least half of the mice analyzed. This indicates that each mouse has its own individual “fingerprint” of antibodies made in response to this curative therapy, and that some epitopes and proteins are clearly more immunogenic because of their co-recognition by several immune mice. To our knowledge, this manuscript is the first time that immune sera from mice cured of a cold tumor by combined radio-immunotherapy have ever been systematically compared to naïve sera from those same mice, to identify every linear peptide in the mouse proteome recognized by antibody induced in the process of bearing and rejecting the tumor. The purpose of this manuscript is to present the novel methodology, demonstrate the in-depth ability to probe ~8.5 million peptides, validate the reproducibility of this immune serological recognition of a fraction of those peptides, and confirm these results using 2 separate independent platforms for evaluating how immune sera from these mice recognize some of these same peptides.

## 2 Materials and methods

### 2.1 Mice and *in vivo* tumor treatment

**Figure 1:**
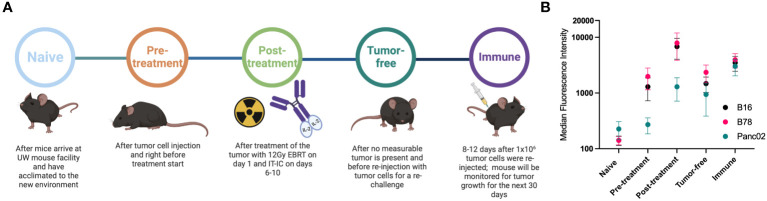
Mice develop antibodies against melanoma tumors throughout treatment. **(A)** Timeline of blood serum collection. C57BL/6 purchased from vendors were allowed to acclimate 1-2 weeks prior to Naïve sample collection and B78 tumor implantation. After measurable tumors were established, Pre-treatment samples were collected prior to initiation of radio-immunotherapy [12 Gy external beam radiotherapy (EBRT) and intratumoral hu14.18-IL2 immunocytokine (IT-IC)]. Following completion of therapy, Post-treatment samples were collected. Tumor-free samples were collected from animals that have no palpable tumors ~30 days following treatment initiation. ~90 days post treatment initiation, these “cured” animals were rechallenged with tumor cells and Immune samples were collected the following week. Schematic created using BioRender. **(B)** Flow cytometric analysis of serum antibody binding to tumor cells. Murine blood serum was incubated with murine tumor cells prior to staining with fluorescently tagged anti-mouse IgG antibodies and flow cytometric analysis. Median fluorescence intensity values corresponding to the timepoints described in A are shown. Serum samples were tested against B16 melanoma (black), B78 melanoma (pink) and Panc02 pancreatic adenocarcinoma (green) murine tumor cell lines. Error bars show standard error of the mean, n=3 mice for each datapoint. Individual mice used were mice A3, A4 and B2 with individual flow cytometry histogram plots shown in **Supplementary Figure 1**. B78 vs B16 vs Panc02 are not statistically significantly different as determined by a two-way Anova (p=0.0675) The post-treatment timepoint shows significantly lower binding for Panc02 than B78 (p=0.0067) and B16 (p=0.0255) in a Tukey’s multiple comparisons test while all other comparisons are not significant.

The treatment model used here was previously described in detail (4, 6, 11). In brief, B78-D14 (B78) cells were injected in C57BL/6 mice. Tumor bearing mice were treated when tumors reached ~ 100 mm^3^ with a combination of 12 Gy local radiotherapy (RT), followed 5 days later with 5 daily intratumoral (IT) injections of the hu14.18-IL2 immunocytokine (IC). Mice that were cured were rechallenged after 90 days with an additional injection of the B78 tumor. Mice that rejected the rechallenge were considered immune (**Figure 1A**). At indicated timepoints (**Figure 1A**), blood via mandibular bleed was collected into BD serum collection tubes and serum was harvested. For select animals a terminal bleed was obtained via cardiac puncture immediately following euthanasia via CO^2^ asphyxiation to obtain larger volumes of serum from immune mice. All experiments and procedures were performed under an animal protocol approved by the University of Wisconsin’s Institutional Animal Care and Use Committee (Protocol number: M005984-R01). A list of all serum samples from individual naïve and immune mice, used to generate the data presented in this report is included as **Supplementary Table 1**. Naïve mice were chosen as the control to represent a healthy mouse background and to allow for detection of anti-cancer antibodies generated against the cancer as well as generated in response to the therapy which should both be present in the immune sample.

### 2.2 Tumor cells

B78-D14 [“B78”, obtained from Ralph Reisfeld (Scripps Research Institute) in 2002] melanoma is a poorly immunogenic cell line derived from B78-H1 cells, which were originally derived from B16 melanoma (8, 9, 22). B78-D14 cells lack melanin, but were transfected with functional GD2/GD3 synthase to express the disialoganglioside GD2 (8, 22), which is overexpressed on the surface of many human tumors including melanoma (23). B16-F10 melanoma was obtained from American Type Culture Collection (ATCC) in 2005. The murine pancreatic ductal adenocarcinoma cell line Panc02 was purchased from ATCC. Panc02, B78 and B16 cells were grown *in vitro* in RPMI-1640 (Mediatech) supplemented with 10% FBS, 2mMol L-glutamine, 100U/ml penicillin, and 100μg/ml streptomycin. Mycoplasma testing via PCR was routinely performed and only mycoplasma negative cell lines were used.

### 2.3 Flow cytometry

0.5x10^6^ cells of B16, Panc02 or B78 were used per tube and incubated with 1μl of Fc block (TONBO biosciences, clone: 2.4G2, catalog # 70-0161-U500) for 5 min prior to adding 1μl of serum for 45 minutes. After incubation, cells were washed with 3ml flow buffer (PBS with 2% FBS) at 300xg and stained with goat anti-mouse IgG-APC (BioLegend, clone Poly4053, catalog # 405308) and rat anti-mouse IgM-PE (ThermoFisher, clone eB121, catalog # 12-5890-82) polyclonal antibodies. Cells were washed again at 300xg for 5min with 3ml flow buffer and resuspended in 50-100μl flow buffer. A drop of DAPI (BioLegend, catalog # 422801) was added to each tube before data was acquired on a ThermoFisher Attune flow cytometer. Data analysis was performed using the software FlowJo version 10.

### 2.4 High-density peptide array

#### 2.4.1 Design of mouse whole proteome peptide microarray

The mouse whole proteome peptide microarray was designed based on the protein set downloaded from UniProt in December of 2018 for C57BL/6 mice (24). The library was generated *in silico* for synthesis on high-density peptide microarrays (Nimble Therapeutics, Madison WI). The library consisted of overlapping 16-mers representing the entire linear continuous mouse proteome tiled at every second amino acid for reviewed proteins and every 4 amino acids for most unreviewed proteins. All redundant (non-unique) peptides were only printed once (resulting in 6,090,593 unique peptides) but later computationally mapped back to all UniProt IDs containing this peptide (resulting in 8,459,970 unique probe IDs). The individual peptides in the library were randomly assigned to positions on the microarray to minimize the impact of spatial biases. Slides were printed as previously described via light-direct array synthesis (25).

#### 2.4.2 Peptide array sample binding

Mouse serum samples were diluted 1:100 in binding buffer (0.01M Tris-Cl, pH 7.4, 1% alkali-soluble casein (as a blocking agent), 0.05% Tween-20). Diluted sample aliquots were bound to arrays overnight for 16–20 hours at 4 °C. After binding, the arrays were washed 3x in wash buffer (1x TBS, 0.05% Tween-20), 10 minutes per wash. Sample binding was detected via goat-anti-mouse IgG Alexa Fluor 647 conjugated polyclonal antibody (Jackson ImmunoResearch, 115-605-071). The secondary antibody was diluted in secondary binding buffer (1x TBS, 1% alkali-soluble casein, 0.05% Tween- 20) and incubated with arrays for 3 hours at room temperature, then washed 3x in wash buffer (10 minutes per wash) and 30 seconds in reagent-grade water. Then the array was washed 2x for 1 minute in 1x TBS and washed once for 30 seconds in reagent-grade water. Fluorescent signal of the secondary antibody was detected by scanning at 635 nm at 2μm resolution and 25% gain. This gain setting was determined based on internal on-array controls to ensure an optimal dynamic range of fluorescence signal intensities. The scanning was performed using a Roche NimbleGen MS 200 micro-array scanner. Subsequently, data were extracted and pre-processed using in-house developed software tool called SlideViewer, and the results were reported as relative fluorescence units.

#### 2.4.3 Peptide array data processing

The datasets generated and analyzed for this study can be found on Zenodo under the following DOI: 10.5281/zenodo.7871565.

For each serum sample, the fluorescence intensity data from a single chip, for each unique peptide, was assayed and processed once; then results from identical peptides redundant to multiple proteins (i.e., were present in more than one protein represented) were restored to each protein. Raw fluorescence intensity signals from primary antibodies binding to peptides on the array, and secondary antibodies with a fluorescent tag binding to primary antibodies were reported. The amount of fluorescence signal is influenced by both the titer and affinity of primary antibodies binding to each peptide sequence.

#### 3.4.4 Data analysis workflow/pipeline of whole proteome data

Detailed bioinformatic/biostatistical data modeling, algorithms, analyses, and graphic presentation methodologies are beyond the scope of this manuscript focusing on the biology and immunology of what is detected using the sera of these immune mice. These issues, and their justification/rationale are presented in detail in a separate manuscript (26). In short, raw data (spatially corrected pre-processed data from Nimble Therapeutics) from the whole proteome peptide array was log2 transformed, quantile normalized, and smoothed using a sliding average mean window across the protein location of +/-8aa.

### 3.5 JPT peptide array

Samples were sent to JPT (JPT innovative Peptide Solutions, Berlin, Germany) and a custom designed PepStar Multiwell Peptide Microarray was performed following manufacturers protocol using a manufacturing process based on SPOT synthesis as described previously (27, 28). The JPT Multiwell Peptide Microarray allowed for 189 peptides per well with 20 wells per slide. One peptide per well was used as an internal control from JPT. Peptides were chosen based on different criteria from the high-density peptide array results, as described in the results section. We included 376 16-mer peptides with a range of signal from the high-density peptide array data and tested those on the same serum samples as well as additional serum samples from immune and naïve mice at a dilution of 1:100. Raw data obtained by JPT for these analyses were sent to us for further analysis and processing. Data were reported as relative fluorescence units.

### 3.6 Peptide ELISA

For the peptide ELISA, 16 separate JPT BioTides™ Biotinylated Peptides were purchased containing a TTDS-linker and biotinylation at the N-terminus. The peptides were generated using the same SPOT synthesis as the larger peptide array (27). Peptides were synthesized from C- to N-terminus ensuring that only full-length peptides will have a biotin at the N-terminus. Coating of streptavidin plates was performed per manufacturers instruction with a 250-fold dilution of lyophilized BioTide peptides. ELISA was performed according to JPTs peptide ELISA protocol with the adaptation to a 384 well plate instead of the standard 96 well plate to conserve on serum samples. Neutravidin coated 384 well plates by ThermoScientific (#15400) were used. Stop solution was added after a 30-minute TMB incubation. Plates were read at regular intervals during TMB substrate incubation (reads at 655 nm) and right after addition of stop solution (reads at 450 nm). Optical density values, read immediately after adding the stop solution were used to analyze results. A standard curve based on a p53-specific 16-mer peptide sequence and a commercially available antibody was used at 12 distinct concentrations, alongside a repeat serum sample with known reactivity, to ensure reproducibility across plates and allow for normalization if deemed necessary, i.e. the control sample values were outside a 10% range of consistency between separate plates.

### 3.7 Choosing of peptides for JPT and ELISA

Peptides for JPT analysis were chosen before the second dataset of whole proteome data using the high-density peptide array was generated and analyzed. 376 peptides were chosen based on different signal strength and reactivity to sample types. In more detail, peptides were chosen based on high signal (>500) in at least one immune sample and low to no signal (<20) in naïve samples. Some peptides were included because they shared, or partially shared, amino acid sequences. Others were chosen because they exhibited no antibody binding in any tested sample or because they had binding in every single sample. We also chose some peptides that exhibited low to medium signal.

For ELISA validation we chose a total of 16 peptides, 2 peptides without any reactivity in any tested sample, 5 peptides based on good correlation between JPT and whole-proteome results and 12 peptides (3 of which were also included in the JPT to whole-proteome correlation category) that showed significant binding in at least 3 immune samples in the moderate (or restrictive) category. We also confirmed that the expected binding sequence within each of these 16 peptides did not have the same, or very similar, sequence to those of any of the other peptides in this group of 16 peptides, to help ensure that each peptide would be identifying relatively distinct antibodies. We also chose 10 random peptides from all unique peptides from the array utilizing a random number generator.

### 3.8 Statistical analysis

#### 3.8.1 Peptide array processing

Data from 13 total unique serum samples were tested in the high-density microarray: 5 from naïve mice, 6 immune samples were obtained from mice following their RT+ IC induced cure from their initial B78 tumor, and then 8-12 days following their rechallenge with another injection of B78 tumor; and two samples (replicates) were obtained from separate mice after a 2^nd^ rechallenge injection of B78 tumor (**Figure 1A**). Matched naïve and immune serum samples were used from 3 separate mice (A3, A4, B2), an additional immune serum sample without a matched naïve control was used (mouse C4). In addition, 2 immune samples (mice AC5 and PD1) were run separately and included in 2 immune pools (each immune pool contained sera from 4 immune mice. The naïve pools contained naïve samples from 4 mice each, including AC5 and PD1 (more detail can be found in **Supplementary Table 1**). These 13 serum samples were assayed for antibody binding to 6,090,593 unique sequence probes mapped to a total of 8,459,970 unique probe IDs (due to redundancies in tiling across protein sequences and using a mixed tiling of either 2aa or 4aa across each protein), or a total of 53,640 individual proteins. Using spatially corrected processed data from Nimble Therapeutics, the data were log2 transformed, quantile normalized, and further processed using a sliding average mean window across the protein location of +/-8aa.

HERON (Hierarchical antibody binding Epitopes and pROteins from liNear peptides) is a methodology we have developed, used, and recently reported (26) to determine thresholds for calling antibody binding at the probe, epitope (consecutive probes), and protein level for each sample using meta-analyses methods to summarize binding across subjects in the immune condition. Briefly, 1) a global p-value was calculated using a z-test for each probe signal using all sample and probe values, and 2) a differential p-value was calculated using a t-test between the average of the naïve samples and each individual immune sample. The global p-value and differential p-value for each immune sample were then combined using the Wilkinson’s max meta p-value method (29). After correcting for false discoveries using the Benjamini-Hochberg (BH) method (30), the individual probes for each immune sample are considered bound by antibodies if their false discovery rates (FDR) are below a threshold. Epitope regions were identified by applying the skater algorithm (31) to identify groups of antibody-bound probes (spatially and across subjects), and epitope meta p-values were calculated using the Wilkinson’s max method on the 2^nd^ highest probe p-value (32). Protein p-values were calculated using Wilkinson’s min (or Tippet’s) method (33). After correcting the epitope and protein p-values using the BH algorithm, the epitope and protein sample calls were made using an FDR cutoff. To avoid prioritization of peptides that may be due to spurious noise, singleton probe and epitope calls without calls of neighboring probes, or if the singleton call was not present in repeat immune samples, it was removed. The number of samples that were bound by antibodies for each probe, epitope, and protein were tabulated as K of N statistics (K = # of samples with antibody binding; N = total # of samples).

#### 3.8.2 ICC score

Statistical analysis was conducted using R (v. 4.1.1; R Core Team 2021) and the packages ‘lme4’ (v. 1.1.27.1 (34); and ‘specr’ (v. 0.2.1 (35); for computing intraclass correlation coefficients (ICC). To analyze the agreement in the high-density whole-proteome, JPT, and ELISA instrument readings among selected peptides, log-transformed readings/intensity was modeled and compared using linear mixed-effects models, in which individual samples and the instruments were modeled, respectively, as random effects, while tumor stage and peptide, when applicable, were modeled as fixed effects. Intra-mouse correlation and intra-instrument correlation were accommodated via random intercepts. The ICC was computed from the instrument random effect to estimate their share of variance in the log-transformed readings. An ICC of 0-0.5 is considered poor reliability; 0.5-0.75 is considered moderate reliability, 0.75-0.9 are considered good reliability; 0.9-1 are considered excellent reliability.

#### 3.8.3 Linear regression/r^2^ score

Simple linear regression was performed using GraphPad Prism (Version 9.5.0, 2022) and r^2^ values were reported. These values were used to describe how predictive the high-density peptide data for the same sample is of the JPT peptide data. The closer the r2 value is to 1, the more predictive the high-density peptide array value is of the JPT value.

#### 3.8.4 Test of proportions

A test of proportions was used to compare the portion of positive reactivity between different peptide groups at a threshold of 2 or higher for OD readings. A p-value of 0.05 was considered statistically significant. The proportion of reactivity in the randomly selected peptides (1 of 200 with OD >=2) were found to be significantly less reactive than the HERON validation set (3+ FDR 0.05, 48 of 240 with OD>=2), respectively.

#### 3.8.5 Hypergeometric testing

The ELISA data replicates were first averaged together. A threshold of ≥2 O.D. (optical density) units was used to call positive antibody binding for each ELISA data point. For each peptide, the fraction of peptides with antibody binding was calculated for the immune samples in the original and validated ELISA set and for the naïve samples in the validated set. A peptide was considered validated if 25% or more of the respective samples were found to be positive.

To calculate the likelihood of getting *n* antibody-bound (positive) peptides out of a random sample of 14 draws, a hypergeometric distribution was used to calculate the probability of getting at least *x* positive hits out of 14 random draws, where the total pool of peptides tested by high-density array has *K* positive peptides^1^. As the true fraction of positive peptides (*K/*6090593) within the pool of ~6 million possible peptides to test from the high-density array is not known, we simulated the calculated p-value from the hypergeometric using different proposed fractions of the total positive peptides within the whole set of ~6 million peptides tested by Nimble.

## 4 Results

### 4.1 Mice bearing B78 tumors elicit a humoral immune response with RT+ IC treatment

B78 melanoma bearing mice treated with RT + IC + anti-CTLA-4 generated an antibody response to surface proteins on the B78 (or B16) tumor cells that was measurable at day 22 post tumor implantation (11). To further investigate these antibody responses and to ensure that the RT+IC treatment alone (without added anti-CTLA-4) can elicit a similar antibody response, we collected serum at multiple times before, during and after successful RT+IC treatment of B78-bearing mice (**Figure 1A**). Serum was collected from mice at the following timepoints: before tumor cells were implanted (Naïve); once tumors reached treatment size but prior to treatment (Pre-treatment); within a week of mice completing the RT+IC regimen (Post-treatment) when the tumors were regressing but still present; weeks later after mice were deemed tumor-free and prior to a rechallenge (Tumor-free); and 8-12 days after subcutaneous rechallenge with injection of B78 cells, ~90 days after treatment and >30 days after the mice were tumor free (Immune). At this point, a strong memory response was demonstrated based on the rejection of the rechallenged B78 tumors. These mice were monitored for an additional 5 weeks to ensure complete tumor clearance of the re-engrafted B78 tumor, proving that these mice were immune to B78. Our prior published results in the same setting showed that these mice cured of B78 by this regimen not only rejected a rechallenge of B78, they also rejected a rechallenge of the immunologically similar parent line (B16), but did not reject a rechallenge with the immunologically distinct, syngeneic, Panc02 pancreatic cancer line. These findings demonstrated that these responses were tumor specific (4).

Using flow cytometry, we tested the serum from each of these timepoints for IgG antibody binding to B78 cells. We observed the presence of endogenous anti-tumor antibodies against B78 in tumor-bearing mice starting at the pre-treatment timepoint, with increasing levels of antibody detected by flow cytometry at all subsequent timepoints (**Figure 1B**). To determine the specificity of these anti-tumor antibodies, we also incubated these serum samples with B16 melanoma cells, the parental line to B78 that is GD2 negative as well as a separate syngeneic pancreatic adenocarcinoma cell line, Panc02. Serum antibodies showed recognition of B16 to a very similar degree as to B78 and a lower recognition of Panc02 (**Figure 1B**). Recognition of Panc02 cells by these serum samples might reflect some shared surface antigens between B78 and Panc02 and possibly other cell lines. A part of this enhanced binding could also be caused by the serum of these mice in the later timepoints being hyper-immune and containing multi-fold immunoglobulins in comparison to naïve serum (36) as well as ageing. The data presented in **Figure 1** are the summed flow cytometry results for 3 of the 6 mice studied subsequently in the high-density peptide array, described below; individual mice showed slight variations in the strength of the responses to these 3 tumor lines at different timepoints (**Supplementary Figure 1**).

### 4.2 Whole proteome peptide array results are reliable and repeatable at high signal levels

To investigate what these antibodies are recognizing on the tumor cells, we used a whole proteome peptide array to profile antibody recognition comparing serum from the naïve vs. the immune timepoints (as shown in **Figure 1A**). We selected naïve mice as the control group to represent a healthy mouse background. This choice enabled us to detect antibodies generated against the cancer itself, as well as antibodies generated in response to the therapy. Both types of antibodies were expected to be present in the immune sample. First, we compared the mean signal (processed and raw) for each of the 6 mice against all 8.46 x10^6^ peptides, referred to as probes with unique probe IDs for each individual sample (**Supplementary Table 2**). The vast majority of probes give a signal near the baseline, using either naïve or immune sera, while a small fraction of probes (0.037-0.092%) give raw signals 100-1000 fold higher than baseline. Even so, more of the probes (detailed numerically in the next paragraph) have even stronger signals in the immune sera than in the naïve sera, such that the mean raw score of all immune samples is greater than the mean raw score of all naïve samples (**Supplementary Table 2**: comparing rows 9-18 to rows 2-6, p-value 0.0294 for unpaired t-test). Our hypothesis was that some specific peptides would show significantly higher binding in immune samples compared to naïve samples. Overall, since we are measuring antibody responses to all native peptides within the mouse proteome, we did expect to see antibody binding to some of these peptides in naïve as well as immune samples as previously seen by Hulett et al. in 2018 (37).

**Figure 2:**
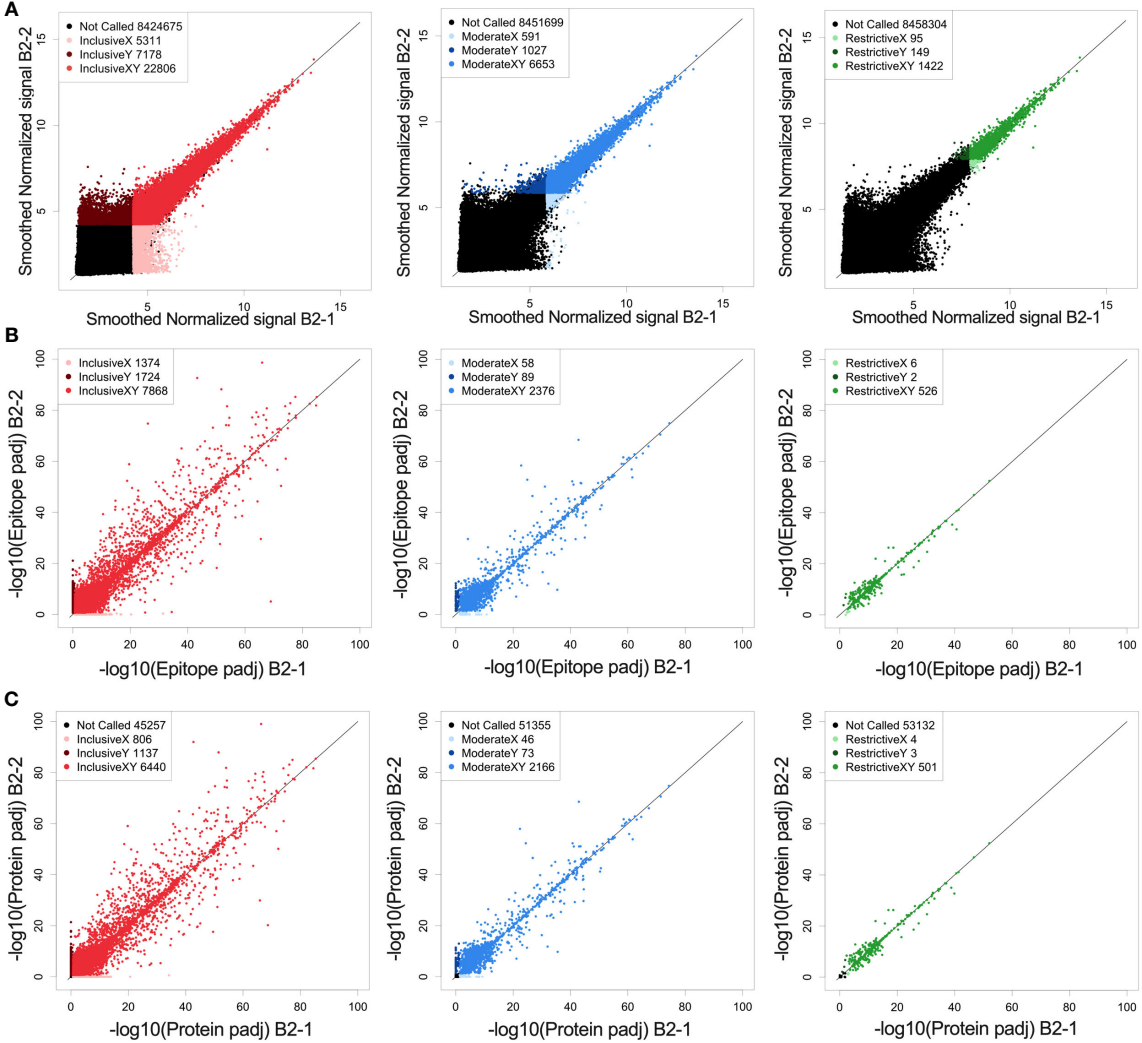
Reproducibility and reliability of probe, epitope, and protein calls. **(A)** Correlation of fluorescence intensity values from two separate whole-proteome microarray chips run one day apart (B2-1 and B2-2) on the same immune-serum from one representative mouse. **(A)** Each dot represents the log transformed processed raw array data for an individual called peptide. These peptides were then separated into 3 categories based on their signal strength: restrictive (highest signal, >10xSD above the mean), moderate (>6xSD above the mean), and inclusive (>3x SD above the mean) based on the statistical significance for each value above the mean signal strength for all peptides. Lighter colored dots represent peptide only called in the B2-1 assay, darker colored dots represent peptides only called in the B2-2 assay, for its respective category (Red: inclusive, Blue: moderate and green: restrictive). For each graph, the black dots are those peptides that were not called for that graph (at the indicated signal strength level) either in the B2-1 or the B2-2 assay. In the inclusive category (signal >3xSD greater than the mean) 76% of probes recognized in sample B2-2 are also recognized in sample B2-1. In the moderate category (signal >6xSD greater than the mean) 87% of probes recognized in sample B2-2 are also recognized in sample B2-1 and in the restrictive category (Signal > 10xSD greater than the mean) 90% of probes recognized in sample B2-2 are also recognized in sample B2-1. **(B)** Scatter plots of epitope-level data based on the peptide data shown in **(A)**, again segmented into restrictive, moderate, and inclusive rankings. Epitopes were identified based on overlapping consecutive recognized peptides and values plotted based on the -log10 p-values. Lighter colored dots represent epitopes only called for sample B2-1, darker colored dots represent epitopes only called for sample B2-2, for its respective category. B-left, showing inclusive category with 72% of epitopes co-recognized by both samples, B-middle showing moderate category with 94% of epitopes co-recognized by both samples and B-right showing restrictive category with 98%. of epitopes co-recognized by both samples. **(C)** Scatter plots of predicted protein-level data based on the peptide and epitope data shown in **(A, B)**, segmented into restrictive, moderate, and inclusive rankings. Proteins were identified by combining epitope data and generating a protein p-value and values plotted based on the -log10 p-values. Lighter colored dots represent proteins only called in replicate sample B2-1, darker colored dots represent proteins only called in replicate sample B2-2, for its respective category. C-left showing inclusive category with 77% of proteins co-recognized by both samples, C-middle showing moderate category with 95% of proteins co-recognized by both samples and C-right showing restrictive category with 99% of proteins co-recognized by both samples. For **(A–C)** the numbers in the legend box within each figure indicate the number of dots in each category. A detailed comparison of each category and its % concordance can be seen in **Supplementary Table 2**.

Prior to identifying high binding peptides recognized by individual or multiple mice, we evaluated how reproducible the signal strength is using this high-density peptide array system. Serum samples from an individual immune mouse (mouse B2), taken after rejection of rechallenge (the immune timepoint in **Figure 1A**) was divided and separate aliquots were analyzed in the same array assay, on independent “chips”, each quantifying the binding signal against all 8.46x10^6^ 16-mer peptides. The paired values for each of these peptides, in the 2 parallel samples are plotted on the X and Y axes in **Figure 2A**. We first looked at all peptides with significantly higher binding than the mean overall signal, defined as a signal that is larger than three standard deviations (SDs) above the mean (inclusive) (left panel of **Figure 2A**, red). A similar analysis was performed for 2 separate aliquots of immune serum from the same blood sample, but from a separate immune mouse (mouse PD1), that was performed on 2 separate identical chips against all peptides, but the analyses were run on separate days, about one year apart (**Supplementary Figure 2A**). However, when looking at all probes that fit these criteria and plotting replicate sample results against each other (**Figure 2A** and **Supplementary Figure 2A**), we noted that at 3SDs, we found a number of probes where one of the values was >3SD from the mean but the replicate sample gave a result that was ≤3SD from the mean. In **Figure 2A**, the number of probes in red, (i.e., seen by both replicates, and designated in the legend box as “InclusiveXY”), corresponds to 65% of the non-black probes with 35% of the non-black probes comprised of the lighter and darker red probes (**Figure 2A**). Because one of the values for these lighter and darker red probes was not >3SD from the mean, these values were not consistent or reproducible, therefore less reliable to call as antibody binding to the peptide.

To enhance reliability and reproducibility of results, we increased the signal strength criteria to ≥6SDs above the mean (middle panel of **Figure 2A**, “moderate”, in blue) which included the top ~0.1% of peptides compared to the top 0.4% of peptides at 3SDs in the inclusive category.

A further increase in signal strength criteria created the “restrictive” category (right panel of **Figure 2A**, “restrictive”, green) which was set at 10SDs. This restrictive category showed the best reproducibility between the 2 paired samples on a peptide level which includes only 0.02% of all peptides. The specific numbers of probes in each category, for each of the paired serum samples for **Figures 2** and **Supplementary Figure 2** are provided in **Supplementary Table 3**.

**Supplementary Table 2** highlights the stark difference for all analyzed samples between the immune and naïve samples, when focusing on peptides called in the moderate and restrictive categories using immune sera; namely comparing high mean signal intensity for the immune samples against the peptides recognized in the moderate or restrictive categories in rows 31-42, with the low signal intensity for naïve samples against those same peptides in rows 21 to 30. However, when comparing overall mean values for naïve or immune samples (rows 2-8 or 9-20), it shows similarity between samples.

**Figure 3:**
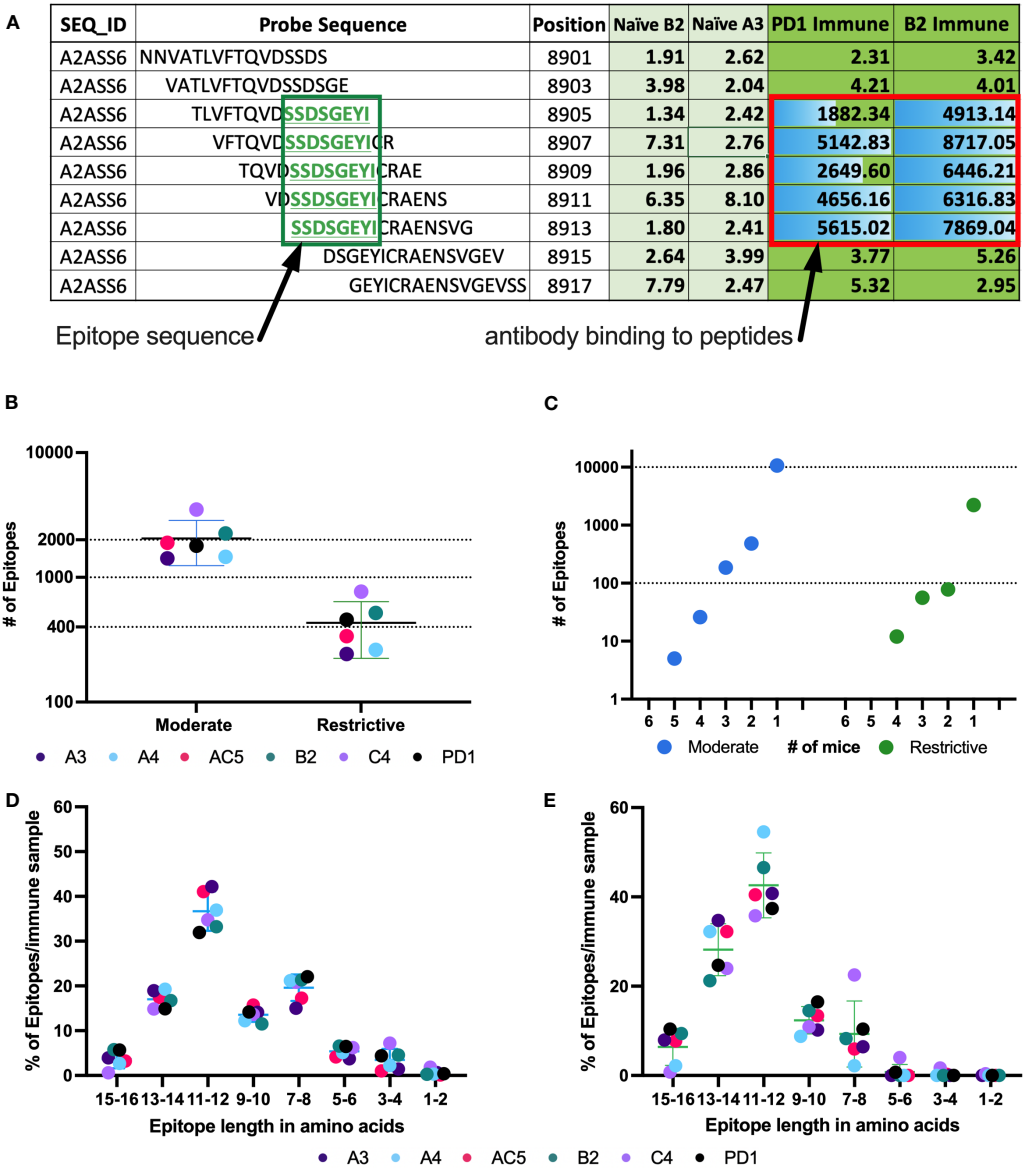
Number of epitopes identified and categorized from mouse whole proteome peptide microarray for all Immune samples. **(A)** Example of raw data highlighting a predicted epitope, defined as a clustered and overlapping antibody binding region in the peptide microarray. A section of the titin protein is shown, with 9 stacked 16-mer peptides, each shifted by 2 aa positions, starting at aa position 8901- 8917. Fluorescence intensity results are shown for each of these 9 16-mer peptides for separate serum samples from 2 naïve mice (naïve B2 and naïve A3) and 2 immune mice (PD1 and B2). Five of the consecutive 16-mers show strong binding by the 2 immune sera, while the other 4 16-mers show very weak binding by all 4 sera shown. The 5 well recognized 16mers each share the 8 sequential aa shown in the green box, indicating a recognized epitope **(B)** Number of moderate and restrictive epitopes identified in the immune samples with significantly higher antibody binding in Immune serum than in naïve serum samples. Each dot represents the number of epitopes in that category, for each of the 6 separate mice tested. [The individual mouse identifications are indicated as separate colors for panels **(B, D, E)**]. **(C)** Number of unique epitopes each recognized by any individual immune mouse, or co-recognized by 2, 3, 4, 5 or 6 Immune mice (of 6 total mice), segmented by cutoff category of moderate and restrictive of the epitopes. Within each category, the single dot plotted above the individual numbers plotted on the X axis indicate the number of epitopes recognized by exactly that number of mice. **(D, E)** Categorization of epitopes by peptide length, based on the clustering as in **(A)**, using data from the moderate **(D)** and restrictive **(E)** category. Above each pair of numbers (i.e.: 1-2, 3-4, etc.) on the X axis are 6 colored dots each indicating the number of epitopes of that aa length recognized by each of the 6 mice tested.

We developed the HERON algorithm to identify consecutive overlapping, reproducible probes with high signal, and categorized the shared aa sequences represented by those highly recognized probes as epitopes based on specified thresholds (**Figure 3A**). The mean signal of an epitope was calculated based on the mean signal of all peptides that comprise the epitope. We again used the categories of moderate and restrictive (based on the single probe calls), but now based on standard deviations as well as false discovery rates (FDRs) to assure that all epitopes with significant signal were counted. Reliability and reproducibility for each of these categories increased significantly by looking at epitopes rather than probes alone and helped eliminate many of the non-reproducible binding events seen only on X or Y axes, but not both (**Figure 2B** and **Supplementary Figure 2B**, **Supplementary Table 3**).

At the protein level, we again were able to see an increase in reliability of called proteins based on signal strength (**Figure 2C**). The stronger a protein was recognized (based on signal strength within each epitope in the protein), the higher the percentage of co-recognition by the 2 replicate serum assays (plotted on the X and Y axes) were found: the inclusive category showing 78%, moderate category showing 95% and restrictive category showing 99% of proteins co-recognized by both replicate samples (**Figure 2C**, **Supplementary Table 3**). When comparing data from the same sample from different runs, the percent of proteins co-recognized by both replicate samples also increased in comparison to probes and epitopes, with 50% in inclusive, 66% in moderate and 75% in restrictive (**Supplementary Figure 2C**, **Supplementary Table 3**). Overall, we see increasing levels of reproducibility (namely co-recognition of the same peptides, epitopes or proteins by the 2 replicate samples evaluated on separate chips on the same day, or on different days), when going from the inclusive to the moderate to the restrictive category. Furthermore, we see increasing levels of reproducibility within each of these 3 categories, when going from peptide to epitope to protein with satisfactory levels of reproducibility and reliability in the moderate and restrictive categories leading us to proceed with these two categories.

### 4.3 Some epitopes are identified by multiple mice

Using consecutive peptides that show high fluorescence signals in immune sera but not in naïve sera enabled us to identify binding epitopes as well as which part of the peptides contained the binding sequence. **Figure 3A** shows at the top an exemplary 16 aa sequence of the protein Titin (UniProt ID A2ASS6), ranging from aa position 8901 to position 8917. Under it are 8 more consecutive 16-mer peptides, each shifted 2 positions to the right from the one above it, thus overlapping with it by 14 amino acids. For this section of Titin, no binding was observed to any of these 9 peptides by the 2 naïve sera tested (A3 & B2). However, strong binding was seen, reflected in high signal numbers, by sera from 2 immune mice, PD1 and B2. The center 5 peptides all show strong binding by both of the immune sera, indicating that the shared 8 aa sequence of these 5 peptides, SSDSGEYI, reflects the antibody binding sequence. The shared 8 aa sequence, recognized in these 5 overlapping peptides, is referred to as an epitope. Overall, using data from the high-density peptide array, we were able to identify an average of 2200 epitopes in the moderate category and just under 500 epitopes in the restrictive category by the immune serum samples from each of the 6 immune mice studied (**Figure 3B**). Of the identified epitopes, many were recognized by only one mouse, while some epitopes were recognized by sera from 2 or more mice, with 5 epitopes being recognized by sera from 5 of the 6 mice in the moderate category (**Figure 3C**). However, with increasing signal strength requirements, these same epitopes were recognized by fewer than 5 mice when using the restrictive category. In the highest binding (restrictive) category, twelve epitopes are each recognized by sera from 4 mice (**Figure 3C**), while 2450 of 2644 epitopes are recognized by only a single mouse (different epitopes for different mice). For the moderate category, 11491 of 12327 epitopes are recognized by only a single mouse. These findings are in line with previous studies looking at protein arrays where the abundant and heterogeneous nature of plasma and serum autoantibodies, regardless of disease status, was discussed (38, 39).

Previous reports stated that an average length for a linear B cell epitope is around 5 to 12 aa (16, 40, 41). Consistent with this, over 90% of epitopes within the moderate and restrictive category are between 7 and 16 aa long (**Figures 3D, E**). Note that a very small fraction of epitopes is identified with a length of 1-2 aa. These small epitopes may be an artifact of the computer algorithm, and most likely suggest that at least two separate antibodies in an individual mouse’s serum are binding to overlapping epitopes in this 1-2 aa region, such that we are actually measuring the overlap of the 2 longer epitopes. However, with the data that we have, it is impossible to determine the start and end for each individual overlapping epitope within the region. In general, we found that epitope length varies slightly across binding strength categories (**Figures 3D, E**).

### 4.4 A greater fraction of proteins than epitopes are bound by sera from multiple mice

**Figure 4:**
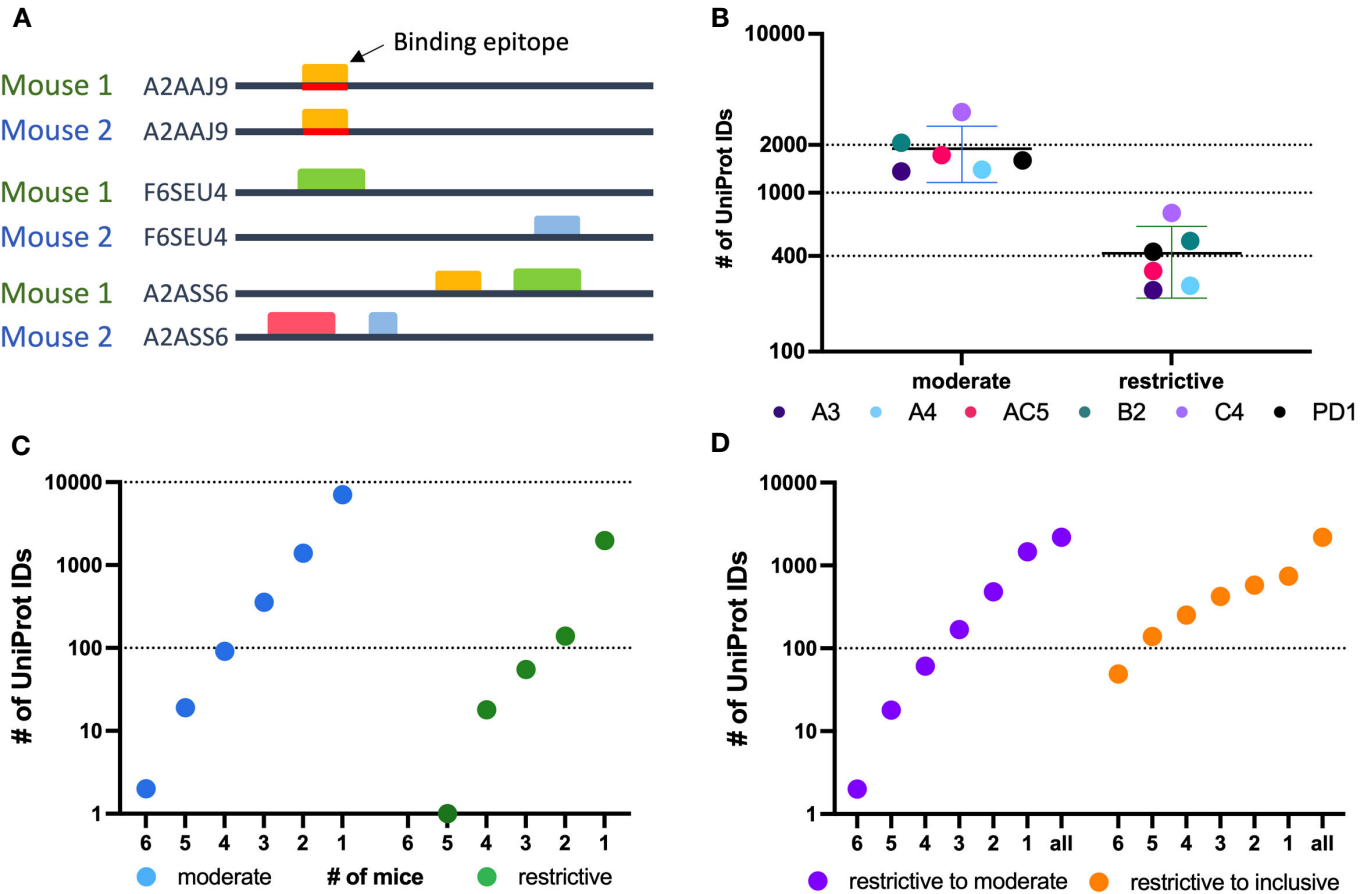
Protein level analysis of Nimble Therapeutics mouse whole proteome peptide microarray data and identified epitopes. **(A)** Schematic showing examples of conditions that can lead to identification of one protein recognized by sera from 2 separate mice, including conditions where the same epitope within the protein is not recognized by antibodies from both mice, even though the protein is recognized by sera from both mice. **(B)** Number of unique UniProt IDs recognized per Immune sample, segmented by the moderate and restrictive categorization of the called protein. Mouse IDs are color-coded to demonstrate similar overall distribution within each category. **(C)** Number of moderate and restrictive unique proteins recognized by at least 1, 2, 3, 4, 5 or 6 of the 6 mice tested. **(D)** Number of proteins identified by the given number of mice on the X axis, where at least one mouse recognized the protein within the restrictive category and the other mice identified that same protein at least in the moderate category (purple) or at least in the inclusive category (orange) in the other samples.

A greater fraction of recognized proteins was bound by sera from multiple mice than were found when evaluating epitopes. This difference in proteins vs. epitopes recognized by multiple mice reflects the different requirements for the determination of recognition of a protein vs. an epitope. For an epitope to be recognized by sera from 2 separate mice, the 2 serum samples need to recognize the same epitope. In contrast, for a protein to be recognized by sera from 2 separate mice, each of the 2 serum samples need to recognize that protein, but not necessarily at the same place on the protein; in other words, if the 2 serum samples recognize distinct epitopes, even at opposite ends of the protein, then these 2 serum samples still recognize that individual protein, as shown schematically in **Figure 4A**. For each of the 6 mice tested, an average of 1963 recognized proteins were in the moderate category as compared to 447 in the restrictive category (**Figure 4B**). However, using sera from multiple mice, 469 proteins were recognized by at least 3 mice within the moderate category, but only 2 proteins were found to be recognized by sera from all 6 mice. In the restrictive category, 74 proteins were recognized by sera from at least 3 of 6 mice, and only 1 protein was recognized by 5 of the 6 mice (**Figure 4C**).

To broaden the criteria for recognition by sera from multiple mice, we focused on all proteins that were detected in the restrictive category by at least one mouse (2188 total unique UniProt IDs) and looked at these UniProt IDs to see if they were detected by sera from any of the other 5 mice using the restrictive to moderate (purple) criteria (**Figure 4D**) to see how many of these mice would recognize these same proteins when the signal strength requirement was loosened. We were able to detect 2 proteins that were now recognized by all 6 mice in this restrictive to moderate category. Overall, 33% of proteins seen by at least one mouse using the restrictive category were seen by 2 or more mice, and 11.4% were seen by 3 or more mice. A similar analysis is also shown for proteins recognized by at least one mouse in the restrictive category, and by other mice using the inclusive (orange) criteria (**Figure 4D**). This showed that 66% of proteins seen strongly by at least one mouse are recognized by two or more immune mice, while 40% are recognized by 3 or more mice. These analyses indicate that there are several proteins recognized by more than one mouse, while the strength of the recognition signal of the peptide array system can vary from mouse to mouse.

### 4.5 Separate peptide ELISA techniques validate whole proteome peptide array data

**Figure 5:**
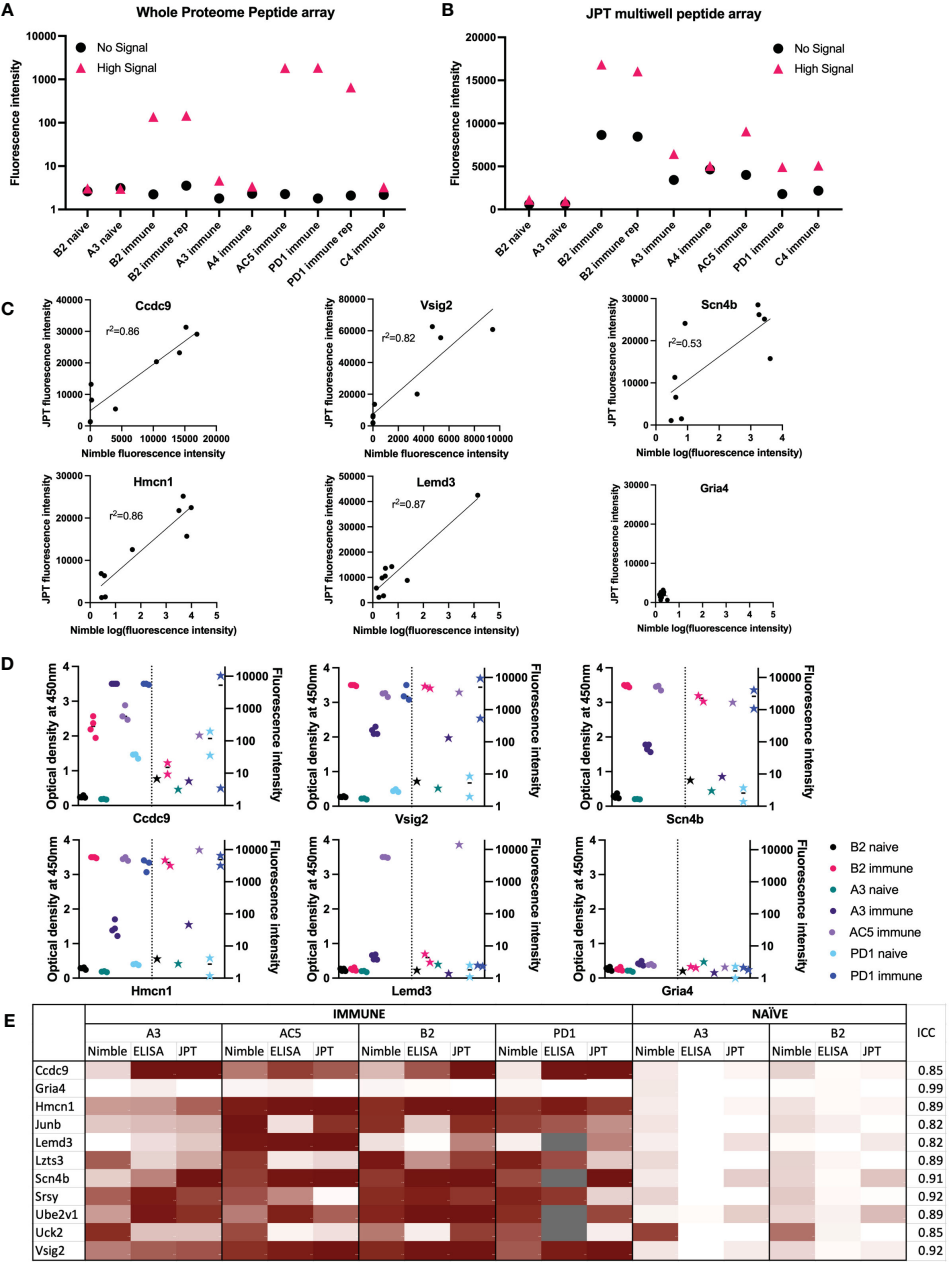
Comparisons of data from Nimble and JPT systems, for the same peptides and serum samples. **(A, B)** Comparison of results using the same 10 serum samples tested in both JPT and whole proteome (Nimble) systems, for 12 peptides selected from the whole proteome data to show no significant signal with any serum samples (naïve or immune) vs. 272 peptides showing a high signal with at least one immune serum sample (A&B). **(A)** Median fluorescence intensity values (from the whole proteome system) for peptides with a high signal (>1000 fluorescence units, >10SD over the mean) in at least 1 immune serum sample that were also tested on the JPT peptide array (pink triangle, high signal, 272 peptides) and on 12 peptides with a signal below 10 fluorescence units in all samples (identified based on the immune and naïve samples from the first whole proteome chipset) (black circle, no signal, 12 peptides) are displayed for 10 serum samples tested in the whole proteome system. **(B)** Median values for the same peptides as shown in A are shown for the same samples (minus the repeat PD1 sample which was only run once on JPT) run on JPT multi-well peptide array. **(C)** JPT (Y-axis) vs. Nimble (whole proteome, X-axis) fluorescence signal data-comparison plots for 6 exemplary peptides from the 11 peptides available to compare all 3 systems as displayed in **(E)** Simple linear regression was performed an the r^2^ value was reported for 5 of the 6 peptides. **(D)** Whole proteome data to ELISA comparison plots for 6 representative peptides (the same as displayed in **(C)** on 7 separate serum samples. For each peptide shown, the left Y axis shows ELISA data as optical density readings, and the right Y axis shows original whole proteome peptide array fluorescence intensity data for the same serum samples on the same peptide. The 7 individual serum samples are displayed in each graph with the same color, ELISA data are shown as circles, whole proteome data are shown as stars. The vertical dotted line separates ELISA from Nimble data. Multiple datapoints [dots (ELISA) or stars (Nimble, whole proteome) for one sample] show replicates. **(E)** Heat map of 11 peptides from 11 different proteins with results from 4 immune serum samples and 2 naïve samples across 3 different peptide binding assays. Results from Nimble whole proteome peptide array as well as JPT peptide array and peptide ELISA were performed on the same serum samples and peptides; results are visualized via heatmap. Eight of the peptides shown were selected based on significant binding by at least 50% of immune serum samples in the whole proteome system. Grey fields indicate no data availability. Scaling for the heatmap was determined individually for each system, using high signal and low/no signal as reference points. White signifies no signal, while dark red indicates high signal within each respective system. ICC: Intraclass correlation coefficient, is the reliability measure of the instrument for that specific peptide accounting for time of treatment and mouse. ICC scores of 0-0.5 show poor reliability, 0.5 - 0.75: Moderate reliability, 0.75 -0.9: Good reliability and 0.9-1 excellent reliability.

After we established HERON, the method used above for the detailed analyses of peptide array data of the proteome recognized by immune sera from mice (and detailed further in a separate companion bioinformatic manuscript (26), we wanted to validate our findings with a separate, independent, antibody detection system for 16-mer aa probes that uses a different technology. For this we used a JPT multi-well peptide array to test 284 16-mer peptides, allowing for testing of a larger number of serum samples. We chose peptides to use in this JPT system based on the results obtained using HERON analysis applied to data from the analyses of the entire proteome, summarized above. We included a small number of peptides that showed no binding in any (naïve and immune) serum samples and also included a larger number of peptides that showed significant level of binding by one or more of the immune serum samples using the data from the proteome analyses from the 6 immune mice tested. The full panel of peptides selected, and the level of their reactivity with naïve and immune serum samples are presented in **Supplementary Table 4**. We used some of the same serum samples that we previously tested on the whole proteome high-density array (**Figure 5A**) to test these 284 peptides on the JPT array (**Figure 5B**). We show the mean reactivity for these same peptides and these same sera using the whole proteome data and the JPT system data (**Figures 5A, B**). Both naïve samples show no binding in either the high signal peptide or no signal peptide groups on the whole proteome peptide array as well as the JPT multi-well peptide array (**Figures 5A, B**). Immune serum samples showed very similar trends, with higher mean signals seen for the high signal peptides than for the no signal peptides. The A3 and A4 immune samples have a low mean signal for the high signal peptides in the whole proteome array as well as JPT. Overall, these results show that Nimble peptide array data can be qualitatively reproduced using an independent JPT multi-well peptide array. Note that the peptides in the Nimble system are biotinylated at the opposite end from that for the JPT peptides, and thereby fixed to the plate at opposite ends; this makes the peptide available to the sera in reverse orientation, thereby partially accounting for some of the discordant results regarding the non-identical recognition patterns for the same peptides in these two systems.

The assessment of responses to some of the individual single peptides tested in both systems, demonstrates a qualitative relationship between the magnitude of responses by individual immune mouse serum samples, when tested on the same peptide in the Nimble and JPT systems (**Figure 5C**). We examined the same peptides recognized by the same serum samples as shown in **Figure 5C** utilizing a separate peptide ELISA system (**Figure 5D**). The ELISA data showed these same serum samples show a qualitatively similar pattern to that seen using the Nimble data for these same peptides. A summary of Nimble to JPT to ELISA comparison for 11 peptides using 4 immune and 2 naïve samples is shown in **Figure 5E**. The overall intraclass correlation coefficient (ICC) for instrument (Nimble, JPT, ELISA), considering peptide, tumor stage, and intra-mouse correlation, was 0.86. At the peptide-level, accounting for naïve vs. immune and intra-mouse correlation, these comparisons show 4 peptides with excellent ICCs (> 0.90: Gria4, Scn4b, Srsy and Vsig2) and 7 with good ICCs (0.75-0.90). None of the tested peptides received a moderate (0.5-0.75) or poor (< 0.5) ICC, thereby demonstrating that these 3 ways of measuring antibody responses are not important sources of variation in the measurement of antibodies to these peptides. Overall, these three assay systems showed similar patterns of response for the peptides we chose to evaluate.

### 4.6 Single peptides follow a similar trend in reactivity as seen with surface staining via flow cytometry

**Figure 6:**
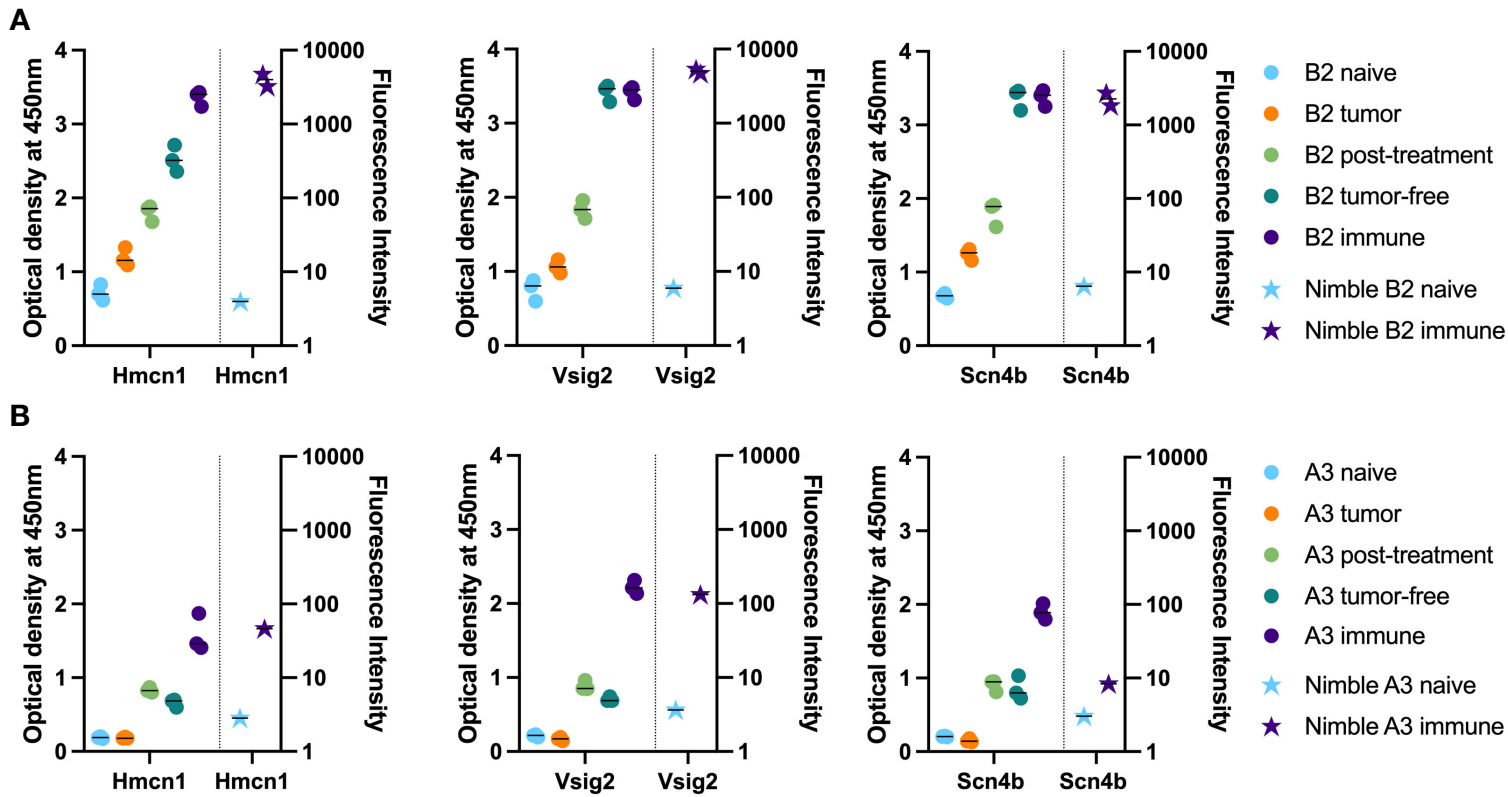
Time-course analysis and validation of Nimble peptide array results via peptide ELISA. **(A, B)** Peptide ELISA of three exemplary peptides (16-mer peptides belonging to Hmcn1, Vsig2 and Scn4b) on all serum collection timepoints shown in **Figure 1** on the indicated separate serum samples from 2 immune mice [B2, top row **(A)** and A3, bottom row **(B)**] are shown as optical density on the left Y axis. Right Y axis displays the corresponding fluorescence intensity from the Nimble Peptide array system for the indicated naïve and immune timepoints. Three separate replicate data points are shown for each serum specimen for each peptide in the ELISA (left Y axis), and 2 replicate data points are shown (at times these overlap) for each serum sample on each peptide for the immune Nimble (whole proteome) data (right Y-axis).

We chose 3 peptides to test all 5 separate timepoints of serum collection shown in **Figure 1** using samples from 2 mice. These peptides were chosen based on high signals for these 3 peptides using most immune samples tested, as well as low signals with most naïve samples tested. **Figure 6A** shows how the level of antibody from mouse B2 towards the specific peptide increases with each subsequent serum sample (as detected by peptide ELISA) until reaching a plateau and then remains at that peak level while **Figure 6B** shows overall lower levels of antibody (as detected by peptide ELISA) for mouse A3 towards the selected peptides. Furthermore, we were able to observe stable antibody concentrations from post-treatment to tumor-free timepoints followed by an increase in antibody in our immune timepoints. In contrast to the flow data reactivity to B16 cells (**Figure 1B**), we were not able to see that the post-treatment timepoint exhibited the highest antibody binding for these three specific peptides.

### 4.7 Validation cohort shows binding to most of the 14 peptides selected for binding in immune samples

**Figure 7:**
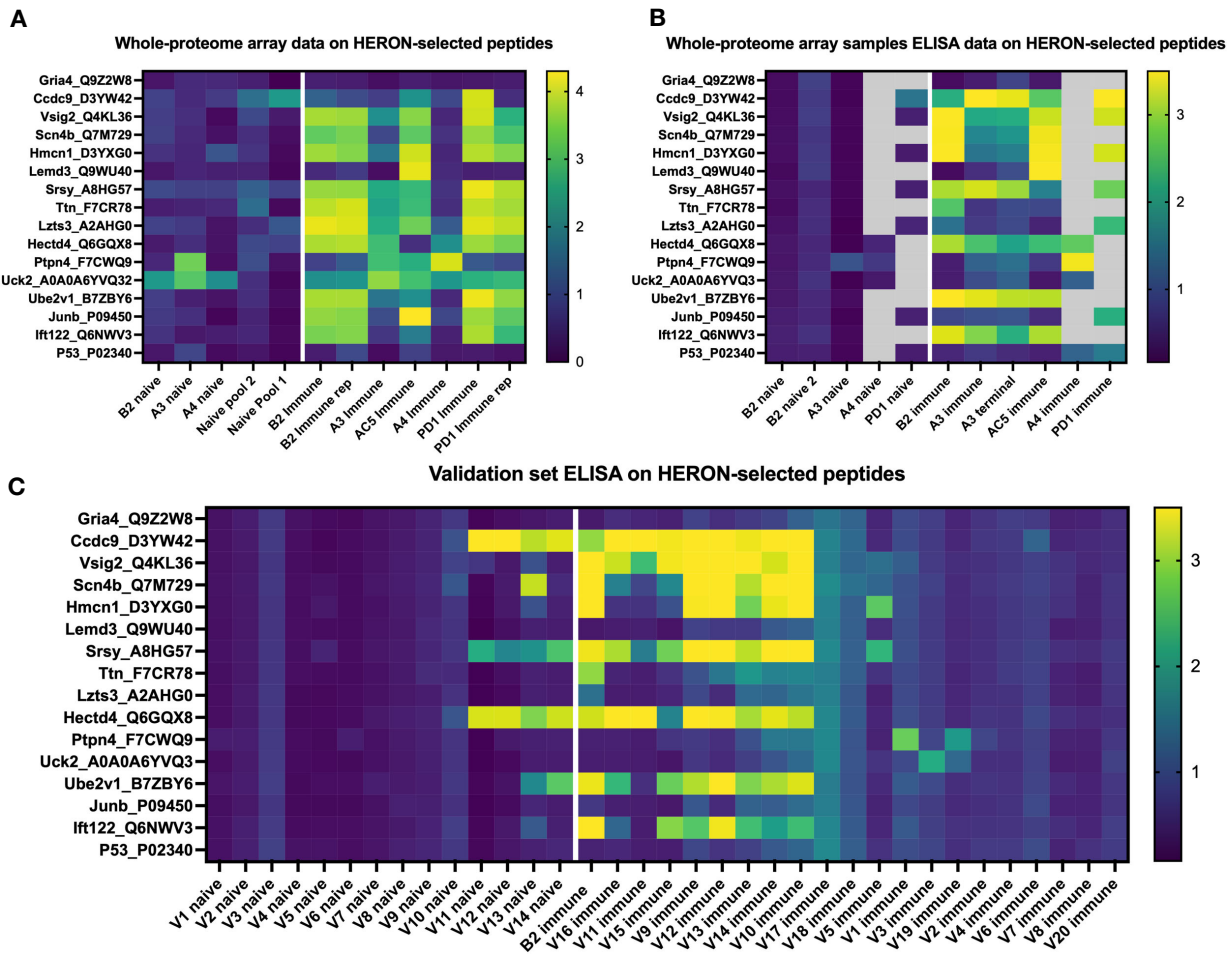
Peptides identified by the whole proteome array are also seen by ELISA testing for the same 6 immune mice, and for a separate validation set of 20 separate immune mice. **(A)** Heatmap of 16 chosen peptides from whole proteome peptide array displaying whole proteome peptide array sample results for 12 serum samples including 2 replicate samples (B2 immune and PD1 immune). White line separates naïve serum samples on left from immune serum samples on right. Twelve of these 16 Peptides were chosen, based on whole proteome data, demonstrating significant binding by serum samples from at least 3 of the 6 immune samples. Two of these peptides were chosen because of selective reactivity in only one or two of the original samples (Lemd3 and Ccdc9). Two of these 16 peptides shown were selected because they exhibited no binding by any of the immune or naïve serum samples tested in the whole proteome system (Gria4 & P53, at the top and bottom of the list shown). Data shown are log10 of the fluorescence units of the peptide array signal. **(B)** Heatmap of ELISA results using the same peptides and serum samples as in **(A)**. Grey areas indicate peptides not tested for the 4 indicated serum samples due to insufficient remaining serum from those samples to enable inclusion in the combinations labelled as grey. Data shown are Optical Density (O.D.) values read at 450 nm length on a scale from 0 to 3.5. **(C)** ELISA data for the same peptides as in A & B but using immune mouse serum samples never tested before from 20 separate mice that have received the same treatment to cure their B78 cancer (together with matched naïve serum samples for 14 of these 20 immune mice). Also included here is a repeat immune serum sample from one of the 6 immune mice used in the original whole proteome samples as an internal control [B2 immune, also shown in whole proteome data in **(A)**, and ELISA data for original whole proteome samples in **(B)**]. Data shown are O.D. values read at 450nm length on a scale from 0 to 3.5.

Lastly, we hypothesized that peptides recognized by immune (but not naïve) sera by 50% or more of our initial cohort of 6 mice would also be seen by sera from additional similarly treated immune mice (as in **Figure 1A**) that were not ever previously tested on any peptide array or ELISA.We selected the following peptides for ELISA testing: 1) 12 well-recognized peptides with binding by sera from at least 3 of 6 immune mice in the moderate category (**Figure 3C**) as tested in the whole proteome peptide array, 2) 2 peptides with significant binding in one or two of the original mice, and 3) 2 peptides without any significant binding in any serum samples from the Nimble system. Of the 6.09 x10^6^ unique peptides tested in the Nimble system for these 6 immune mice, only 316 peptides (0.005%) showed recognition at the moderate level for at least 3 of 6 immune mice. **Figure 7A** shows the original whole proteome data for these 14 recognized and 2 non-recognized peptides for 5 of the original 6 mice. We obtained qualitatively comparable results for these same 16 peptides via ELISA for the same naïve and immune samples that were run on the whole-proteome peptide array (**Figure 7B**). We were able to test the same 5 mice but did not have enough serum available to test all peptides with serum from mice A4 and PD1. In this ELISA, 12 of the 14 previously selected peptides show significant recognition by at least 1 immune mouse, and 8 of the 14 peptides are recognized by at least 2 of these 5 mice. We then proceeded to run these same 14 reactive peptides (and 2 non-recognized peptides) on new naïve and immune samples (previously untested by array or ELISA) collected from mice who had the same B78 tumor and received the same RT + IC therapy as our initially treated mice. ELISA results of the 20 new immune and 14 new matched naïve serum samples are shown in **Figure 7C**. Overall, we were able to show that ~12 of the 20 new immune mice (60%) have antibodies against at least 1 of the 14 reactive peptides with 9 mice showing reactivity to multiple peptides. All new samples exhibited no antibody binding against the 16-mer peptides from Gria4 or P53, just like none of the original samples did in **Figures 7A, B**. However, 6 peptides showed binding to at least 1 naïve sample with 4 of the 14 naïve samples showing some antibody reactivity against at least 1 peptide (**Figure 7C**) (a smaller fraction than observed in the immune samples) which might give less biological importance to these peptides as potential selective cancer targets.

**Figure 8:**
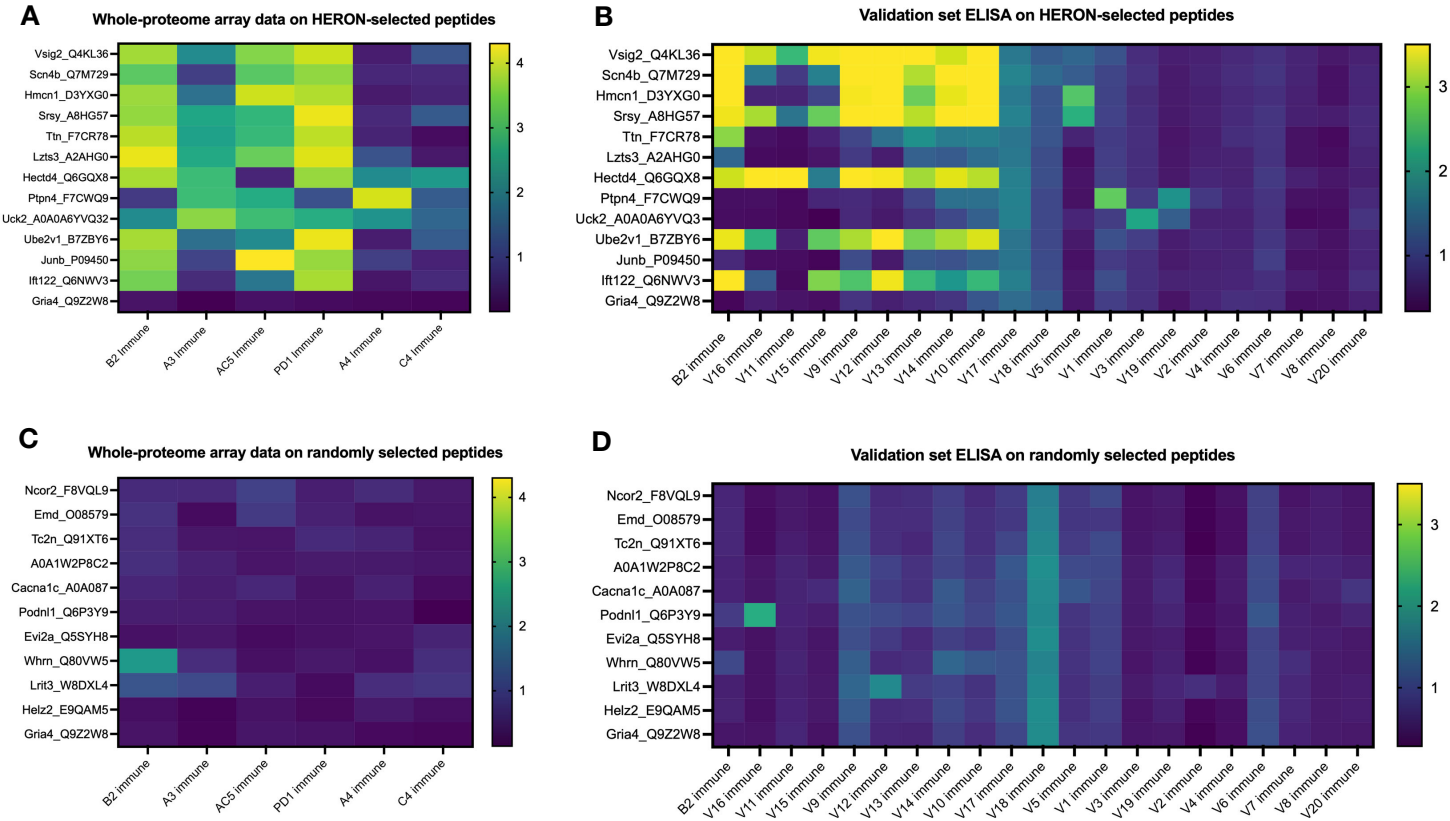
Peptides selected at random from whole proteome array are similarly recognized by ELISA testing for a separate validation set of 20 separate immune mice and show much lower antibody recognition in comparison to HERON-identified peptides present in 50% or more of the original cohort. **(A)** Heatmap of 13 of the 16 peptides highlighted in **Figure 7A** on the whole proteome dataset displaying whole proteome peptide array sample results for 6 immune serum samples (the same 6 immune serum samples used for **Figures 2** & **3**). The peptides included here reflect 12 peptides chosen for strong antibody reactivity in 3 or more of the original 6 mice tested on the whole proteome array. One peptide is included as a negative control peptide that was intentionally selected as a negative control; we have never observed antibody binding to it in any of our original or validation tested samples (Gria4, at the bottom of the list). Data shown are log10 of the fluorescence units of the peptide array signal. **(B)** Heatmap of ELISA data for the same peptides as in A but using immune mouse serum samples not tested on the whole proteome array from 20 separate immune mice that have received the same treatment to cure their B78 cancer. These 20 serum samples and 13 peptides are identical to the 20 immune serum samples and 13 of the 16 peptides shown in **Figure 7C**. Also included here is a repeat immune serum sample from one of the 6 immune mice used in the original whole proteome samples as an internal control (B2 immune, also shown in the whole proteome data in **Figure 7A**, and ELISA data for original whole proteome samples in **Figure 7B**). Data shown are optical density values read at 450nm length on a scale from 0 to 3.5. Scales used for the heatmaps in **(A–D)** are consistent with the scales used in **Figures 7A-C**. **(C)** Heatmap of 11 peptides from the whole proteome dataset displaying whole proteome peptide array sample results for 6 immune serum samples (the same 6 immune serum samples used for **Figures 2** & **3**). Ten of these 11 Peptides were chosen at random utilizing a random number generator out of all probed peptides from the whole proteome array. One peptide is included as a negative control peptide (Gria4, at the bottom of the list). Data shown are log10 of the fluorescence units of the peptide array signal. **(D)** Heatmap of ELISA data for the same peptides as in C but using immune mouse serum samples not tested on the whole proteome array from 20 separate immune mice that have received the same treatment to cure their B78 cancer. These 20 new immune serum samples are identical to the 20 new immune serum samples shown in **Figure 7C**. Also included here is a repeat immune serum sample from one of the 6 immune mice used in the original whole proteome samples as an internal control [B2 immune, also shown in whole proteome data in **(C)**]. Data shown are optical density values read at 450nm length on a scale from 0 to 3.5.

We next chose to compare the large amount of antibody binding observed against the highly recognized peptides tested in **Figure 7**, with a set of randomly selected peptides, to compare the peptide array and ELISA data for the highly recognized, vs. randomly selected peptides. We developed and utilized HERON to choose the set of peptides highly recognized by at least 3 of the 6 mice included on the whole proteome dataset, and present the peptide array and ELISA data for them in **Figures 8A, B**. For comparison, we used a random number generator to pick 10 peptides out of the whole proteome array dataset of 6,090,593 unique peptide sequences. The log-transformed fluorescence intensity values associated with these 10 random peptides and the negative control Gria4 peptide, used previously from the whole proteome peptide array, are shown in **Figure 8C**. All 10 of these random peptides showed virtually no reactivity with any of the sera from the 6 immune mice tested, except for one peptide (Whrn) that showed low, but detectible, reactivity with the B2 immune sample using the data obtained on the original whole proteome dataset. This one somewhat positive reaction out of the 60 possible combinations of 10 random peptides with 6 serum samples in **Figure 8A** corresponds to 1.7% positive. We used these 10 random peptides to probe the immune serum samples from the same 20 validation set mice utilized in **Figure 7C** for antibody binding by ELISA to any of these randomly selected peptides (**Figure 8D**). We observed moderate antibody binding by one of the 20 validation set immune samples (V16) to one of the 10 tested random peptides (Podnl1). No other validation serum samples showed detectible binding to any of these 10 peptides. Thus, of 200 possible combinations of the 20 serum samples with the 10 randomly selected peptides in **Figure 8B**, only one (0.5%) was positive. Contrasting the relatively absent reactivity of these 20 new validation immune samples to these randomly selected peptides, we now show the relatively strong reactivity of these same 20 validation immune serum samples to the 12 peptides from **Figure 7C** selected utilizing HERON analyses of the original Nimble data, that were recognized by at least 3 of the original 6 immune mice. These data are shown in **Figure 8B**, using a selection of data also shown in **Figure 7C**. Unlike the 1 positive reaction out of 200 possible combinations shown in **Figure 8D**, these same 20 immune serum samples now show 20% positive reactions with these 12 HERON selected peptides (48 reactions with an OD reading of >=2 out of 240 possible combinations = 20%) (**Figure 8B**). This substantial 20% ELISA reactivity of these 20 validation sera to these 12 HERON selected peptides is significantly greater (p< 0.001) from the 0.5% reactivity in the randomly selected peptides.

We acknowledge that our sample size of 10 randomly selected peptides in **Figure 8B** is a small fraction of the over 6 million peptides present on the array. To approach and analyze the ability of the HERON method to identify peptides from the initial Nimble data with the original 6 mice that will show greater than chance reactivity with a new set of immune serum samples, using a calculation that includes a larger number of randomly selected peptides, we employed a model utilizing the hypergeometric distribution (**Supplementary Figure 3**). When calculating the probability of having a quarter of the previously untested mice recognize a specific peptide with a high ELISA threshold (minimum O.D. signal of 2) if the peptides would have been chosen at random using a hypergeometric test (P(X >= 8 given 14 draws out of a pool of ~6 million), the chance of having this occur with a separate set of mice is almost zero (**Supplementary Figure 3**). For example, if we assume that 1% (60,906) of the peptides from the set of the ~6 million unique peptides are reactive, the probability of randomly choosing 14 peptides from the pool of possible peptides and finding that at least 8 of the peptides that are responsive to 25% or more of the mice in the new validation set is 1.9x10^-15^. If we are sampling with replacement from the pool of samples, i.e. using a binomial distribution rather than hypergeometric, the probability is still 2.85x10^-13^. The similar result between the hypergeometric and binomial approach is due to the low likelihood of randomly choosing the same peptide twice amongst the large pool of possible peptides. These analyses indicate that peptides recognized at the moderate level using the Nimble array data for immune sera from 50% of multiple mice are highly likely to be recognized by separate, similarly immunized mice, in a validation set, using ELISA data as a validation system.

Since only 0.45% of peptides tested are recognized at the moderate level by at least one mouse (27639 peptides), and only 0.005% of peptides tested are recognized at the moderate level by 3 or more mice (316 peptides), the fact that 12 of 20 (60%) of independent immune mice from the validation set are recognizing at least one of the 14 peptides (selected from the 0.005% of peptides recognized in the Nimble system by 3 or more mice) by the ELISA system indicates that these peptides co-recognized by multiple mice in the Nimble system are identifying peptides likely to be recognized by independent (validation set) immune mice.

## 5 Discussion

The aim of this study was to establish a method to utilize a high-density overlapping stacked array of peptides representing the entire C57BL/6 linear proteome to identify the spectrum of all linear peptides from the full mouse proteome that are seen by mice that received curative radio-immunotherapy associated with complete and durable eradication of B78 melanoma tumors with induction of tumor-specific immune memory. We are not able to determine from these studies whether the recognition of any of these antigens by these antibodies may or may not be involved in the initial tumor rejection, or the rejection of the rechallenged tumor demonstrating memory. We have published that these responses are known to be T cell-mediated (4). While we have shown that a memory humoral response is induced (11), we do not have any data indicating that this antibody response is involved in the actual tumor rejection. A separate manuscript from our team (12) is demonstrating that when a TLR4 activator is added to a similar regimen used in this report, then an antibody response can be involved in the anti-tumor rejection response. In this work, we demonstrated the utility of high-density peptide microarrays for profiling the antibody repertoire in immune serum samples by using a proteome-scale peptide microarray representing all proteins in the mouse proteome. This enabled a fine-mapping of all regions of linear epitopes recognized by circulating antibodies induced during the growth and subsequent complete rejection of a syngeneic murine melanoma. Although these whole-proteome peptide microarrays contained peptides representing the proteome, this study is not a complete analysis of the antibody-detected “immunome”, but rather an analysis of the linear native protein-based antibody responses. The length of the 16-mer peptides is a limitation, as conformational (or discontinuous) epitopes may remain undetected and the majority of antibodies produced, target conformational epitopes (42). Any protein modifications like glycosylation or phosphorylation were also not considered, neither for the peptide array as well as the peptide ELISA data. Nevertheless, these proteome-scale peptide microarrays, along with the development and use of HERON analytic methods, provided an in-depth snapshot of the information stored in the linear antibody repertoire of mice immune to B78 melanoma after successful RT+IC therapy.

We were able to achieve improved reliability and reproducibility when considering epitopes rather than single peptide probes (**Figures 2A** vs. **2B**). A couple of factors contribute to this; first, there are many more probes than epitopes in the proteome, giving a larger number of possible mismatches and false-positive hits from error-prone single peptide values from peptide arrays. Second, an individual epitope can be a component of several overlapping probes; our HERON algorithm for detecting epitopes recognized by separate assessments of serum samples, requires a degree of similar recognition of the related epitope containing probes by the 2 samples, but does not require complete identity of probe recognition and signal. This enables higher reproducibility of epitopes recognized with high signals than peptides recognized with high signals when replicate chips are evaluated for separate aliquots of the same immune serum sample (**Figures 2A** vs. **2B**). We observed a higher variability between the samples conducted a year apart (**Supplementary Figure 2**) compared to those performed within a day of each other (**Figure 2**). This variability could be attributed to several factors including potential effects of additional freeze-thaw cycles on the serum sample, as well as potential changes in the equipment used for the peptide array (including camera and laser). Somewhat similarly, when evaluating proteins that are recognized, since a single protein might be recognized by different mice at different regions of the same protein, the number of proteins recognized by 4, 5 or 6 of the 6 immune mice (at moderate and restrictive recognition levels) is somewhat higher than the number of epitopes mutually recognized by 4, 5 or 6 of the 6 immune mice (comparing **Figures 3C** vs. **4C**).

While signal strength may serve as a predictor for peptide binding reliability, it cannot be used as a measure of antibody affinity (40). Signal strength in peptide arrays is determined by many factors, including quality of synthesized peptide, variations in peptide solvation, presence, or absence of high-affinity antibodies as well as presence or absence of multiple lower-affinity antibodies towards the peptide. As seen in **Figure 3B**, the number of recognized epitopes is similar across all 6 immune mice, while the epitopes recognized by individual mice show a large heterogeneity between mice. This heterogeneity in epitopes recognized is demonstrated by the very large number of epitopes recognized by just one mouse (> 10,000 in the moderate category), compared to the substantially smaller number (< 1000 in the moderate category) of epitopes with mutual recognition by any 2 of the 6 immune mice (**Figure 3C**) and only a much smaller fraction of epitopes (~200) mutually recognized by 3 or more (50%) of the 6 immune mice. A large heterogeneity in antibody repertoire between individuals has been shown before in humans (38, 39) and was expected due to the tremendous variation within the V-D-J recombination leading to the specific binding characteristics of an individual antibody generated by a clonally expanded mature B cell.

Interestingly, when validating just a small cohort of 12 peptides, representing ~2.86% of the peptides out of 420 peptides that were recognized by at least 3 of our original 6 mice based on the Nimble system data at a moderate level, we found reactivity to at least one of these peptides in 60% of our validation cohort of 20 separate immune mice (**Figure 7D**). While we did not achieve the same rate of recognition for each individual peptide, having at least one peptide recognized by some of these additional 20 mice supports the biological relevance of these proteins being antibody targets by multiple mice in our system. This biological importance is further supported by the testing of random peptides with immune serum samples from these same 20 additional mice (**Figure 8**) where 10 randomly selected peptides showed only one of the 20 mice recognized just one of the 10 peptides barely above the threshold of an O.D. value of 2, with a mean O.D. value of 2.28, corresponding to 0.05% positive reactions out of 200. In contrast when these same 20 validation immune serum samples were used to recognize the 12 HERON-selected peptides that showed reactivity with at least 3 of the original 6 mice in the Nimble data, 48 out of the 240 possible combinations had an O.D. reading of 2 or higher (20%, p< 0.001 for 1 reaction out of 200 for random peptides vs. 48 reactions out of 240 for the HERON selected peptides). More importantly, because the antibody repertoire is determined by highly variable gene rearrangements of V-D-J immunoglobulin gene components, the antibody repertoires of distinct genetically identical mice have substantial differences. Thus the ability of the HERON method to identify peptides based on their recognition by an initial set of mice using the Nimble data and then demonstrate that these same peptides are subsequently strongly recognized using an independent ELISA assay, on a separate set of previously untested validation immune serum samples, indicates that the peptides (and epitopes) identified by the HERON-method have immunologic importance for these separate validation mice from the same strain immunized to the same B78 tumor using the same radio-immunotherapy regimen. A comparison of HERON to other analyses tools was published by McIlwain et al. (26).

There are some limitations to the current assay configuration to evaluate peptide binding by serum antibodies using a high-density peptide array technology. The assay is set up to provide end-point binding of a complex mixture of antibodies at a single serum dilution. It is difficult to estimate the absolute binding affinity of each antibody clone in the complex sera from such a mixture model. By using different dilutions of known concentrations of well characterized mAbs known to recognize specific epitopes or peptides on the Nimble array, a “standard curve” could be created enabling one to interpolate signals seen with immune sera to the standard curve with the mAb dilutions, allowing calculation of a “mAb concentration equivalent”. We have not pursued this and do not think this has been done yet by others using this technology. By evaluating serial dilutions of multiple different mAbs, at the same concentrations, on this high-density proteome array, one might be able to investigate some general patterns to allow quantitative assessments of binding to elements of the proteome with this technology. Once this knowledge has been acquired, this peptide array might be an optimal way to characterize the binding and specificity/cross-reactivity for new mAbs being developed.

It is also possible that this array misses the antibody target of some clinically important antibody responses. These include antibody reactivity against conformationally determined (or discontinuous) epitopes that are not generated in relatively small 16-mer peptides and make up the majority of targets (42). Second, as the peptides used in this array are strictly 16-mer aa sequences, no glycosylation or phosphorylation is applied to these peptides. Thus, circulating antibodies that recognize differentially glycosylated or phosphorylated peptides would not be detected. Third, some clinically important antibody targets have no peptide component. A major example is the GD2 disialoganglioside, proven to be a clinically important target on neuroblastoma; this glycolipid has no peptide component and is recognized by the Dinutuximab mAb (43), and the hu14.18-IL2 immunocytokine used to cure mice of B78 melanoma in this study (4, 11), and also recognized by circulating antibody in patients immunized with a GD2-containing vaccine (44). This peptide array would not be able to identify antibodies that might have been turned on to such non-peptide antigens, even if they were strongly induced in the process of these mice rejecting, and developing, a memory immune response to these B78 tumors. However, the array would be able to detect antibody binding to peptide mimotopes of such antigens as they are cross-reactive with the non-peptide antigens (45–47). Finally, some of the antigenic targets on tumors that have been recognized by adaptive immunity are mutation-driven neo-antigens, with aa sequences different from that controlled by the inherited germline genome. As each individual tumor will have its own unique set of neoantigens, the detection of antibodies to these neoantigens using this high-density peptide array technology would require independently created proteome arrays to be established for each individual tumor being evaluated. Translating such an approach to clinical testing would be challenging at present.

As such, while other epitope discovery methods are superior in probing more limited numbers of targets to define discontinuous, conformational, phosphorylated, glycosylated, non-peptide, or mutated epitopes and immunodominant responses, this technology appears useful in identifying immunoreactive regions within the entire proteome, not previously considered as potential epitopes, on a large scale.

Beyond the possible utility of identifying biomarkers for effective immune responses induced by cancer immunotherapy, we are hopeful that this technology can be used in profiling antibody responses to many other diseases. This tool was able to detect known and previously unknown protein targets of antibody responses throughout the mouse proteome. This approach could potentially be applied to other cancers to advance diagnostic and cancer vaccine development.

This initial description of the results of our analyses to probe the detection of linear epitopes recognized by sera of mice cured of their B78 tumors, relies on the novel bioinformatic approaches developed to analyze these large data sets, reported separately (26). This current report presents: 1) the immunologic methods used to obtain data and validate it using additional smaller-scale peptide arrays and ELISA systems; 2) the spectrum of peptides, epitopes and proteins recognized; and 3) initial description of what fraction of targets recognized by at least one immune mouse are also recognized by some other mice, despite the highly variable nature of each mouse’s individual B-cell repertoires. Important additional ongoing analyses are still being finalized; these are beyond the scope of this initial report, and will be reported in a second manuscript now in preparation. These additional analyses to be reported separately include characterizing which antigens are recognized by these immune sera and determining their relationship to the B78 melanoma tumor that responded to the radio-immunotherapy in the process of turning on these adaptive antibody responses. These ongoing studies also include identifying which of the proteins recognized by these immune antisera are expressed or over-expressed by the tumor cells themselves, and if expressed by the tumor cells, what is their cellular location (membrane, cytoplasmic or nuclear). Furthermore, even though these antibodies we have focused on were not seen in naïve mice, and were thus possibly induced by bearing the tumor, and responding to the radio-immunotherapy, given the very large number of proteins recognized by these immune sera (~10,000 separate proteins recognized by the immune responses of just 6 immune mice, as shown in **Figure 4C**), it seems very unlikely that all of these are selectively expressed by the tumor cells and not normal tissues. As such these antibodies induced in these mice by implanting and successfully treating these tumors may also reflect antibodies that can recognize proteins from normal tissues and may thereby be considered “auto-antibodies”. Such auto-antibodies may be the mechanism behind auto-immune, paraneoplastic syndromes seen frequently in patients with cancer (48, 49). One sign of autoimmunity we have observed in our mice is vitiligo in the location of treatment (data not shown, as this will be presented in detail in the separate manuscript currently in preparation). As vitiligo is linked to the immune system attacking melanocytes, some of which are not melanoma cells, and thus could be a form of autoimmunity, it does suggest that some of the peptides and proteins we observe as targets for increased recognition by immune sera may be related to autoimmunity during this response. Finally, even though multiple distinct proteins are recognized by these immune sera, might some of these antibodies be recognizing shared or similar amino acid sequences on these distinct proteins, reflecting possible immune cross-reactivity of similar antibodies to shared epitopes on seemingly distinct proteins? These issues are now being pursued and will be presented in a subsequent separate report (50).

In summary, this work shows that peptide array technology can be used to detect a large spectrum of linear peptide-based epitopes, recognized by antibodies- in sera from mice immune to B78 tumors through RT+IC treatment. While we saw a large heterogeneity between individual mice, some proteins were strongly recognized by sera from multiple immune mice and may potentially be of importance in achieving immunity to the cancer, or as a biomarker of a potent adaptive response to the cancer. This same type of workflow could be applied to other types of cancer or other diseases as well as to the analyses of patients that have received effective immunotherapy associated with a clear immune-mediated anti-tumor response to their cancer to evaluate the equivalent spectrum of antibody-recognized human tumor proteins. Some work, using this peptide array technology, in this cancer realm, and in analyses of autoimmunity and anti-viral immunity has been reported and more is in preparation (51–54).

## Data availability statement

The original contributions presented in the study are included in the article/**Supplementary Material**, further inquiries can be directed to the corresponding author.

## Ethics statement

The animal study was approved by University of Wisconsin’s Institutional Animal Care and Use Committee. The study was conducted in accordance with the local legislation and institutional requirements.

## Author contributions

AH designed the experiments, collected the data, designed, and performed the analysis, interpreted the data, generated the figures, and drafted the manuscript for this study. SM designed and performed the analysis, generated figures, and assisted in writing and editing the manuscript. NM, AX & DM assisted in data collection and analysis and editing the manuscript. KT, TL and KK helped in the analysis of experiments and editing the manuscript. RP, BG & JP assisted in design and execution of experiments and editing of the manuscript. MH, AF & NT assisted in collecting the data and editing the manuscript. JH and ZM assisted in designing the experiments and editing the manuscript. AE assisted in designing the experiments and interpreting the data as well as editing the manuscript. PS and IO assisted in designing the experiments and analysis, interpreting the data, and editing the manuscript. All authors contributed to the article and approved the submitted version.

## Footnotes

1 To simplify our explanation, we use the binomial distribution, which assumes the probability is the same for every trial (assumes replacement): if there are 10000 positive peptides within the pool of total possible peptides (6000000), the probability of getting k=8 positive peptides out of n=14 selected peptides (if all peptides are equally likely) is p=((6000000 – 10000)/6000000)^k^. The probability of 14-k failures is (1-p)^(14-k)^. However, there are (14/*k*) different ways of distributing k=8 successes in a sequence of n=14 trials, so $P(X=8)=\frac{14!}{9!6!}p^{8}{\left(1-p\right)}^{6}$. Finally, we calculate the sum of the probabilities using 8 to 14 peptides to obtain the cumulative distribution function P(X >= 8).
