# Supplementary material for: Antibody landscape of C57BL/6 mice cured of B78 melanoma via a combined radiation and immunocytokine immunotherapy regimen
Source: Front Immunol. 2023 Nov 23;14:1221155. doi: 10.3389/fimmu.2023.1221155 (PMC10701281; doi:10.3389/fimmu.2023.1221155)
Supplement: Supplementary file 1 [file DataSheet_1.pdf]

Supplemental Figure 1

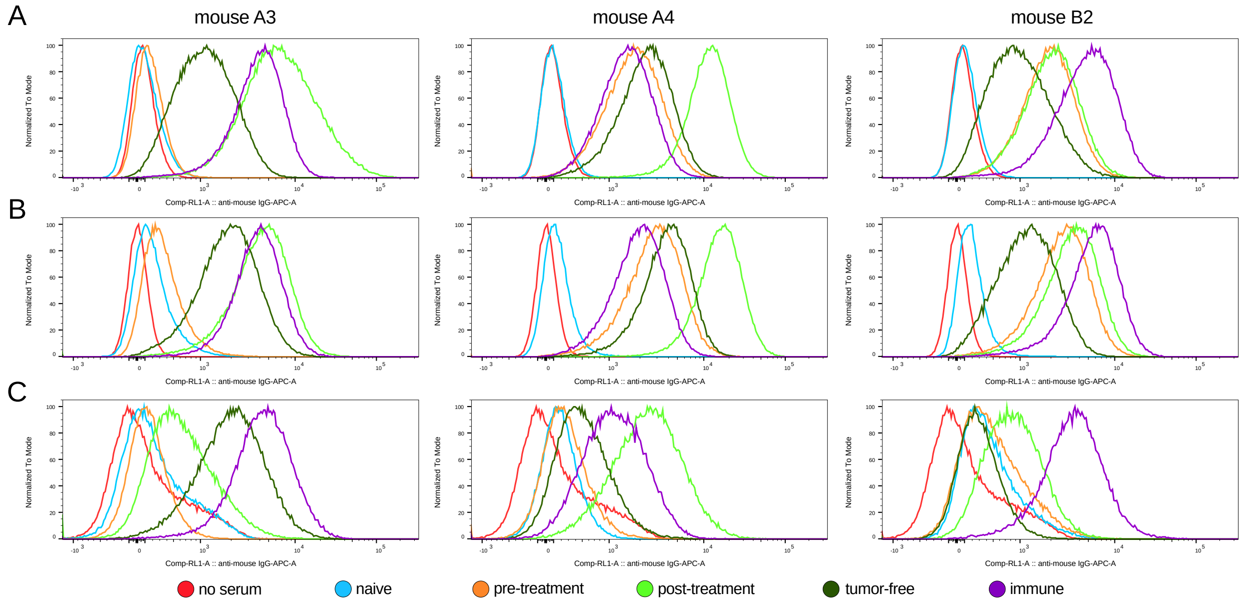

Supplemental Figure 1: Histograms of IgG binding to tumor cells for three individual mice (A3, A4 and B2) on 3 different tumor cell lines.

Timepoints correspond to the sample collection timeline in Figure 1A. **A:** Binding of serum antibodies to B16 tumor cells as measured via flow cytometry. **B:** Binding of serum antibodies to B78 tumor cells as measured via flow cytometry. **C:** Binding of serum antibodies to Panc02 tumor cells as measured via flow cytometry. Data are shown as fluorescence intensities detected in the red channel measuring fluorescence signal for APC. The samples for each individual mouse are normalized to mode to enable comparison between the different time points for each mouse.

Supplemental Figure 2

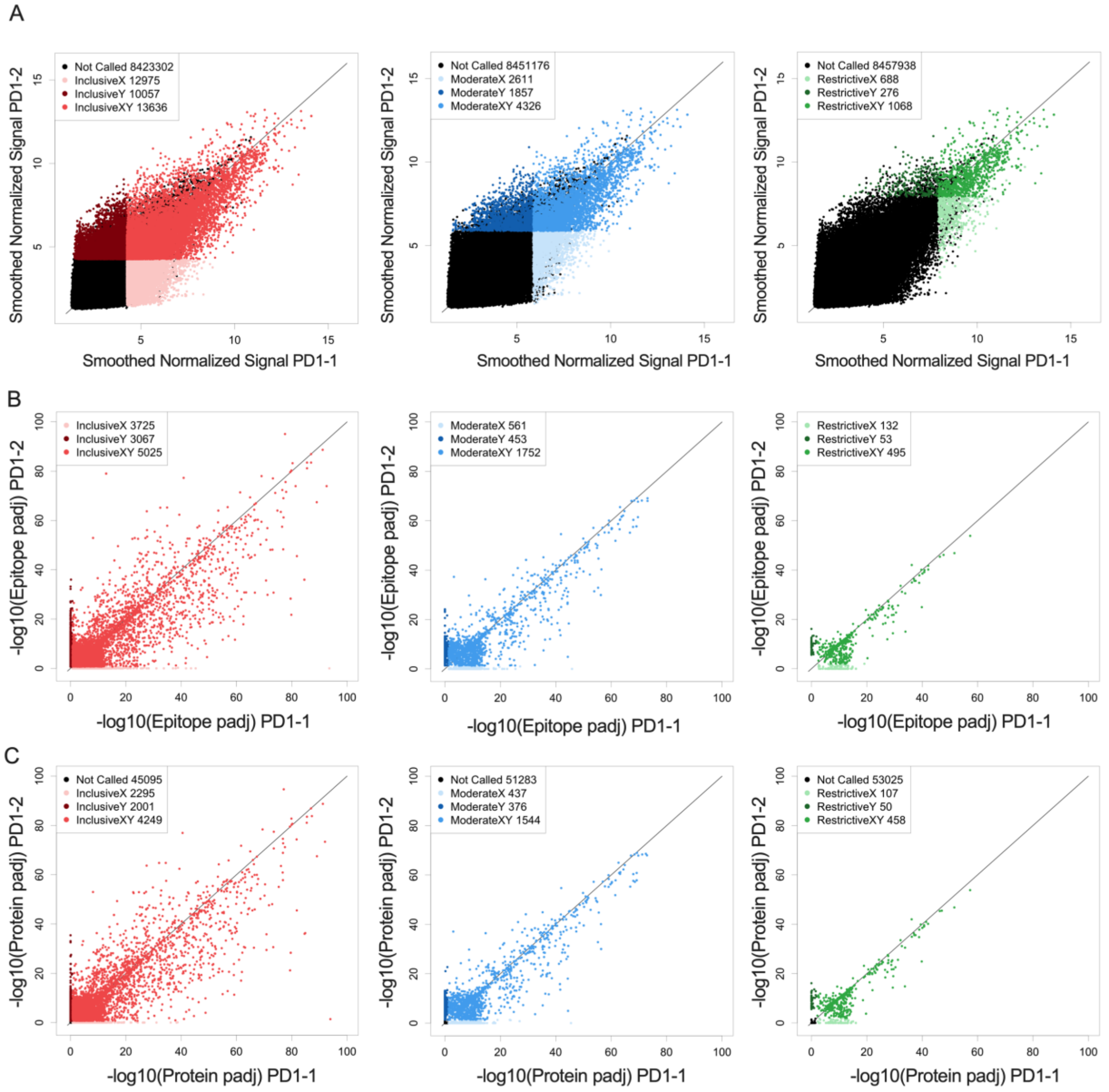

Supplemental Figure 2: Reproducibility and reliability of probe, epitope, and protein calls

A-C: Reliability of Peptide array: Called peptides were separated in 3 categories [left panel inclusive; middle panel moderate; right panel restrictive] based on signal strength of the bound peptides. **A:** Cryopreserved serum samples from the same Immune blood sample from mouse PD1, were tested independently (sample PD1-1 and PD1-2) on identical whole proteome chips, in assays that were performed 1 year apart. These showed high correlation between signal strength and repeatability of results after data pre-processing on a probe level. Shown are the log transformed processed raw peptide array data generated in relative fluorescence units. In the inclusive category (signal >3xSD greater than the mean) 58% of probes recognized in sample PD1-2 are also recognized in sample PD1-1. In the moderate category (signal >6xSD greater than the mean) 70%

of probes recognized in sample PD1-2 are also recognized in sample PD1-1 and in the restrictive category (Signal > 10xSD greater than the mean) 80% of probes recognized in sample PD1-2 are also recognized in sample PD1-1. **B:** Scatter plot of the same serum sample from **A** looking at epitope level data instead of individual peptides. Plotted are the -log10 values of the epitope p values for each epitope recognized in sample PD1-1 and sample PD1-2. Graphs are split up into each category, B-left, showing inclusive category with 43% of epitopes co-recognized by both samples, B-middle showing moderate category with 63% of epitopes co-recognized by both samples and B-right showing restrictive category with 73%. of epitopes co-recognized by both samples, **C:** Scatter plot of the same serum sample from A & B looking at protein level data instead of individual peptides or epitopes. Plotted are the -log10 values of the protein p values for each protein recognized in sample PD1-1 and sample PD1-2. Graphs are split up into each category, C-left showing inclusive category with 50% of proteins co-recognized by both samples, C-middle showing moderate category with 66% of proteins co-recognized by both samples and C-right showing restrictive category with 75% of proteins co-recognized by both samples.

Supplemental Figure 3

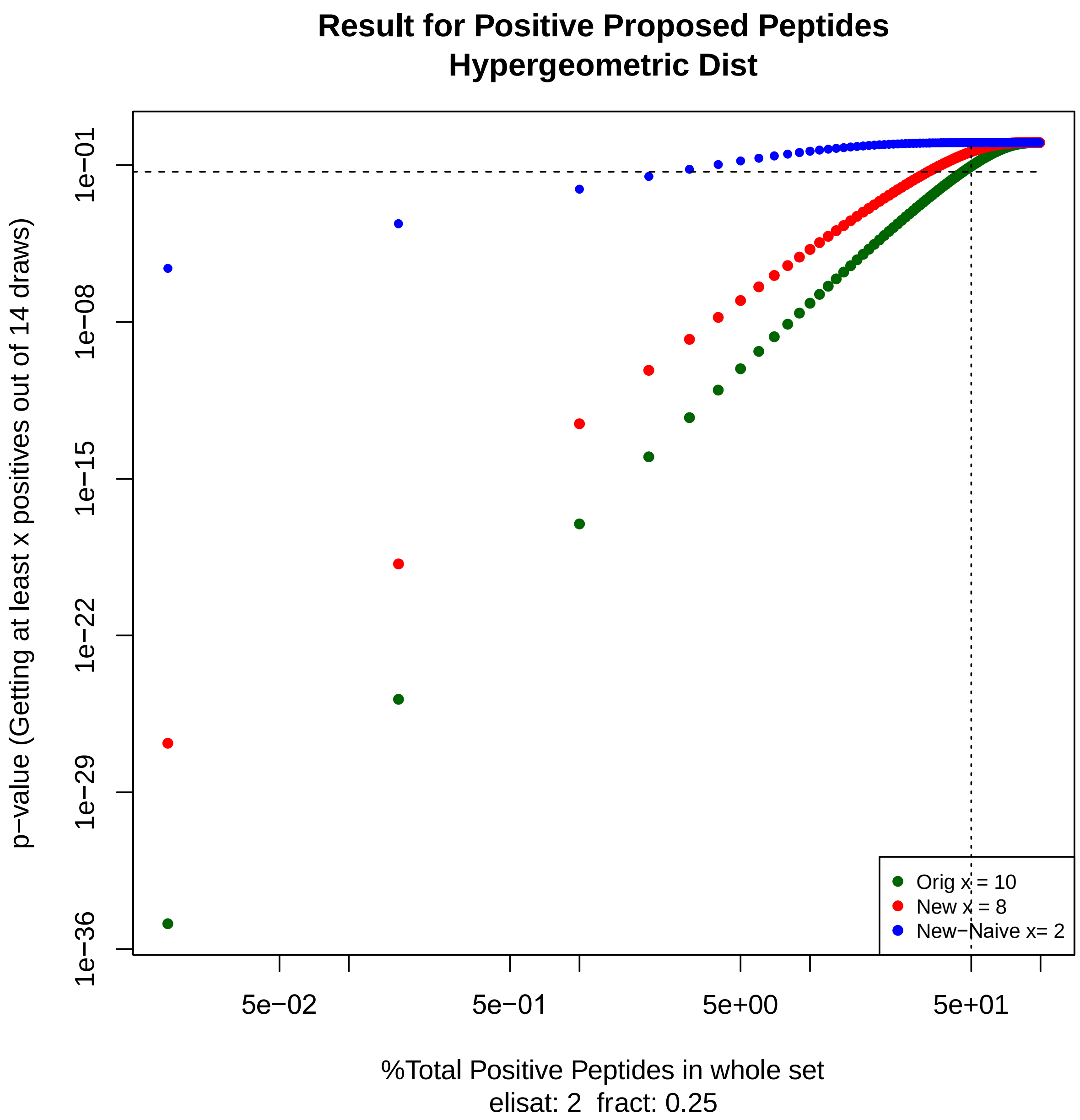

Supplemental Figure 3: Plot of the hypergeometric p-value of getting at least *x* or more positives out of a sample of 14 randomly chosen peptides versus the % of proposed total peptides in the whole set of ~6.5 million unique peptides. Green - original ELISA results on immune mice, Red - validation ELISA results on immune mice, Blue - validation ELISA results on Naive mice. Both the red and green line show very significant p-values for up to almost 50% of peptides of the 6.5million unique peptides giving a positive signal. However, looking at the Naïve samples a significant p-Value is only reached by a maximum of 5% of positive peptides in this simulation using values derived from the Nimble and ELISA data generated.

Supplemental Table 1: List of serum samples used across Nimble Peptide arrays, JPT multi-well peptide array and peptide ELISA with data displayed in this manuscript. Identifiers refer to individual mouse IDs, used for naïve as well as immune (noted in column labeled status). Samples marked as (Pool) on Nimble whole proteome array 1 consisted of 4 separate serum samples each. naïve pool 1 and naïve pool 2 each contained naïve samples from AC5 and PD1 . These were array chips run with pooled samples where it was not possible to distinguish which serum sample caused each specific antibody binding. Samples were used a 1:200 concentration for each specific sample, 1:50 concentration for overall serum concentration in the corresponding array chip. Pooled immune samples were excluded for most analysis purposes, pooled naive samples were used to determine peptides recognized by naïve mice and could be partially matched with immune samples AC5 and PD1.

Identifier	Status	Nimble whole proteome array 1	Nimble whole proteome array 2	JPT multiwell peptide array	ELISA
AC5	naïve (as pool)	X			
	immune	X		X	X
PD1	naïve (as pool)	X			X
	immune	X	X	X	X
B2	naïve		X	X	X
	immune		X	X	X
A3	naïve		X	X	X
	immune		X	X	X
C4	immune		X	X	
A4	naïve		X		X
	immune		X	X	X
naïve pool 1 (AC5, PD1, PD4, PD5)	naive	X			
naïve pool 2 (AC5, PD2, AC1, AB4)	naive	X			
immune pool 1 (AC5, PD1, AB3, PD4)	immune	X			
immune pool 2 (AC5, PD1, CS6, CS4)	immune	X			
Validation cohort for ELISA only:					
V1	naïve				X
	immune				X
V2	naïve				X
	immune				X
V3	naïve				X
	immune				X
V4	naïve				X
	immune				X
V5	naïve				X
	immune				X
V6	naïve				X
	immune				X
V7	naïve				X
	immune				X
V8	naïve				X
	immune				X
V9	naïve				X
	immune				X
V10	naïve				X
	immune				X
V11	naïve				X
	immune				X
V12	naïve				X
	immune				X
V13	naïve				X
	immune				X
V14	naïve				X
	immune				X
V15	immune				X
V16	immune				X
V17	immune				X
V18	immune				X
V19	immune				X
V20	immune				X

Supplemental Table 2:
Overview of raw and processed mean signal strength (and SD) and mean raw signal strength for all serum samples for all peptides tested, separated by naïve vs. immune status. Row 1 shows signal strength for all tested samples. Rows 2 and 3 show results of 2 separate pools of naïve sera from 4 separate mice each. Rows 4-6 show samples from 3 individual naïve mice, row 7 shows all naïve samples averaged together [including the pooled samples (rows 2-6)], row 8 shows all individual mouse naïve samples averaged (rows 4, 5 & 6) excluding pooled naïve samples (rows 2, 3). Rows 9 and 10 show results of 2 separate pools of 4 immune mice in each pool. Rows 11-18 show results for 8 individual immune serum samples, including immune samples from the same 3 individual naïve mice (B2, A3 and A4), tested in rows 4-6. Samples B2_1 and B2_2 and PD1_1 and PD1_2 are the same serum aliquots tested on separate peptide array slides and are described in Figure 2 & supplemental Figure 2. Row 19 shows all immune samples averaged together including pooled samples (rows 9-18); row 20 shows all individual mouse immune samples averaged together, leaving out pooled samples (rows 11-18). Rows 31 to 42 show all immune samples individually, showing processed and raw mean intensity signals only for all peptides selected by HERON on that sample in the moderate recognition group (rows 31-36) or in the restrictive recognition group (rows 37-42). Rows 21 to 30 show average signal of the indicated individual naïve samples when looking at all peptides that were called by HERON on at least one immune sample in either the moderate (rows 21 to 25) or restrictive category (rows 26 to 30). The increase in mean signal is clearly visible in the processed as well as raw data for immune samples in the moderate and restrictive category (rows 31-42) while naïve samples did not differ much when comparing all peptides vs. moderate or restrictive category peptides as determined by at least 1 immune sample call.

#	timepoint	peptides		processed (quantile normalized, smoothed and log transformed)		raw data	# of peptides 100-fold over mean	% 100-fold over mean
			Sample names	Mean	SD	Mean		
1			global (all)	1.99015	0.45211	5.31622		
2	naïve	all peptides	naive_pool 1	1.98849	0.44278	2.35624	7424	0.08775445
3			naive_pool 2	1.99010	0.42613	4.61029	3148	0.03721053
4			naive_B2	1.98964	0.46667	5.77037	4204	0.04969285
5			naive_A3	1.98976	0.43953	4.92331	5306	0.06271890
6			naive_A4	1.99079	0.44973	3.60680	4908	0.05801439
7			Naive_and_pool	1.98976	0.44517	4.25340		
8			Naive_no_pool	1.99006	0.45211	4.76683		
9	immune		immune_pool 1	1.99053	0.46747	5.12063	6043	0.07143051
10			immune_pool 2	1.98965	0.51326	7.31156	5994	0.07085132
11			immune_PD1_1	1.99023	0.45930	4.55581	7830	0.09255352
12			immune_PD1_2	1.99039	0.44836	4.54346	5983	0.07072129
13			immune_AC5	1.98988	0.46046	6.68841	4315	0.05100491
14			immune_B2_1	1.99092	0.47201	6.63859	6088	0.07196243
15			immune_B2_2	1.99075	0.47703	8.46648	5607	0.06627683
16			immune_A3	1.99061	0.44896	4.51709	5706	0.06744705
17			immune_A4	1.99049	0.46811	5.84747	5382	0.06361725
18			immune_C4	1.99072	0.48346	8.05630	7075	0.08362914
19			Immune_and_pool	1.99038	0.46212	6.20545		
20			Immune_no_pool	1.99047	0.45212	6.20191		
21	naïve	all peptides called in moderate immune	naive pool 1 moderate	2.06853	0.52834	3.39948		
22			naive pool 2 moderate	2.11466	0.57600	6.72079		
23			naïve B2 moderate	2.16509	0.66693	9.61581		
24			naïve A3 moderate	2.15960	0.77961	7.21459		
25			naïve A4 moderate	2.10941	0.60592	5.47932		
26		all peptides called in restrictive immune	naïve pool 1 restrictive	2.10591	0.61448	3.88472		
27			naïve pool 2 restrictive	2.15214	0.68374	8.00851		
28			naïve B2 restrictive	2.19721	0.69202	10.18719		
29			naïve A3 restrictive	2.15316	0.65682	13.47541		
30			naïve A4 restrictive	2.11747	0.60719	5.14340		
31	immune	moderate only	immune_PD1_moderate	7.35379	1.32881	946.81228		
32			immune_AC5_moderate	7.08060	1.12767	1074.86461		
33			immune_B2_moderate	7.19672	1.22372	1064.68637		
34			immune_A3_moderate	7.11368	1.20509	769.64641		
35			immune_A4_moderate	7.15830	1.19106	853.85522		
36			immune_C4_moderate	7.21668	1.34456	2177.49914		
37		restrictive only	immune_PD1_restrictive	9.23298	1.13716	2129.10943		
38			immune_AC5_restrictive	9.03314	0.99102	3254.34157		
39			immune_B2_restrictive	9.06303	1.02728	2762.35977		
40			immune_A3_restrictive	9.08933	1.04502	2023.97308		
41			immune_A4_restrictive	8.88492	0.90688	1732.05080		
42			immune_C4_restrictive	9.27342	1.25595	6623.23231		

Supplemental Table 3:

Values corresponding to Figure 2 and supplemental Figure 2 and percentage calculations based on the respective called probes, epitopes, or proteins for serum samples from the same mice at the same timepoints (using the same cut-offs as shown in Fig. 2 and Suppl. Fig. 2) either run within a day of each other (for mouse B2 in Fig. 2) or a year apart (for mouse PD1, in Supplemental Figure 2). In addition: calculations comparing serum from 2 different mice tested in the same run (B2 vs repeat of PD1) show that different mice demonstrate co-recognition of a small fraction of the samples seen by those same individual mice. Calculations for % were done as following: overall=X only +Y only + X&Y; % overall=(overall/all probes)x100; % X&Y=(X&Y/all probes)x100; % of called (X)=(X&Y/(X only + X&Y))/100; % of called (Y)=(X&Y/(Y only + X&Y))x100; % of called X&Y=(X&Y/overall)x100.

	Sample	X (.1) only	Y (.2) only	X&Y	overall	all probes	not called	% overall	% X&Y	% of called (X)	% of called (Y)	% of called X&Y
Probe	B2 Z3	5311	7178	22806	35295	8459970	8424675	0.417	0.270	81.11	76.06	64.62
	PD1 Z3	12975	10057	13636	36668	8459970	8423302	0.433	0.161	51.24	57.55	37.19
	B2 Z6	591	1027	6653	8271	8459970	8451699	0.098	0.079	91.84	86.63	80.44
	PD1 Z6	2610	1857	4326	8793	8459970	8451177	0.104	0.051	62.37	69.97	49.20
	B2 Z10	95	149	1422	1666	8459970	8458304	0.020	0.017	93.74	90.52	85.35
	PD1 Z10	688	276	1068	2032	8459970	8457938	0.024	0.013	60.82	79.46	52.56
	B2vsPD1 (same run, different mice)											
	Z3	25536	21112	2581	49229	8459970	8410741	0.582	0.031	9.18	10.89	5.24
	Z6	6408	5347	836	12591	8459970	8447379	0.149	0.010	11.54	13.52	6.64
	Z10	1355	1128	216	2699	8459970	8457271	0.032	0.003	13.75	16.07	8.00
Epitope	B2 Z3	1374	1724	7868	10966					85.13	82.03	71.75
	PD1 Z3	3725	3067	5025	11817					57.43	62.10	42.52
	B2 Z6	58	89	2376	2523					97.62	96.39	94.17
	PD1 Z6	561	453	1752	2766					75.75	79.46	63.34
	B2 Z10	6	2	526	534					98.87	99.62	98.50
	PD1 Z10	132	53	495	680					78.95	90.33	72.79
	B2vsPD1 (same run, different mice)											
	Z3	8036	6886	1206	16128					13.05	14.90	7.48
	Z6	2434	1844	361	4639					12.92	16.37	7.78
	Z10	427	443	105	975					19.74	19.16	10.77
Protein	B2 Z3	806	1137	6440	8383	53640	45257	15.628	12.006	88.88	84.99	76.82
	PD1 Z3	2295	2001	4249	8545	53640	45095	15.930	7.921	64.93	67.98	49.72
	B2 Z6	45	73	2166	2284	53639	51355	4.258	4.038	97.96	96.74	94.83
	PD1 Z6	437	375	1544	2356	53639	51283	4.392	2.879	77.94	80.46	65.53
	B2 Z10	4	3	501	508	53640	53132	0.947	0.934	99.21	99.40	98.62
	PD1 Z10	107	50	458	615	53640	53025	1.147	0.854	81.06	90.16	74.47
	B2vsPD1 (same run, different mice)											
	Z3	5058	4062	2188	11308	53640	42332	21.081	4.079	30.20	35.01	19.35
	Z6	1767	1475	445	3687	53640	49953	6.874	0.830	20.12	23.18	12.07
	Z10	404	407	101	912	53640	52728	1.700	0.188	20.00	19.88	11.07

Supplemental Table 4:
284 peptides tested on JPT, and Nimble platform used in Figure 5 in no or high signal category. Data provided for all samples from Nimble and JPT shown in Figure 5. Categories were chosen based on Nimble whole proteome array 1 and then tested on JPT and Nimble whole proteome array 2. All data from JPT multi-well peptide array runs and matched Nimble peptides on all samples can be found in Supplemental Data 1. This table can be found in the supplemental excel sheet. labelled "Supplementary Table 4 and Supplemental Data File 1".

Supplemental Data File 1:

376 peptides tested on JPT and Nimble platform. Data provided for all samples from Nimble and JPT runs performed. Categories were chosen based on Nimble whole proteome array 1 and then tested on JPT and Nimble whole proteome array 2. This table can be found in the supplemental excel sheet, labelled "Supplementary Table 4 and Supplemental Data File 1".
